# Identification of Novel Gene Signatures using Next-Generation Sequencing Data from COVID-19 Infection Models: Focus on Neuro-COVID and Potential Therapeutics

**DOI:** 10.3389/fphar.2021.688227

**Published:** 2021-08-31

**Authors:** Peter Natesan Pushparaj, Angham Abdulrahman Abdulkareem, Muhammad Imran Naseer

**Affiliations:** ^1^ Center of Excellence in Genomic Medicine Research, Faculty of Applied Medical Sciences, King Abdulaziz University, Jeddah, Saudi Arabia; ^2^ Department of Medical Laboratory Technology, Faculty of Applied Medical Sciences, King Abdulaziz University, Jeddah, Saudi Arabia; ^3^ Department of Biochemistry, Faculty of Science, King Abdulaziz University, Jeddah, Saudi Arabia

**Keywords:** SARS-CoV-2, COVID-19, Neuro-COVID, bronchial epithelium, cerebrospinal fluid, RNA sequencing, next-generation knowledge discovery platforms, therapeutics

## Abstract

SARS-CoV-2 is the causative agent for coronavirus disease-19 (COVID-19) and belongs to the family Coronaviridae that causes sickness varying from the common cold to more severe illnesses such as severe acute respiratory syndrome, sudden stroke, neurological complications (Neuro-COVID), multiple organ failure, and mortality in some patients. The gene expression profiles of COVID-19 infection models can be used to decipher potential therapeutics for COVID-19 and related pathologies, such as Neuro-COVID. Here, we used the raw RNA-seq reads (Single-End) in quadruplicates derived using Illumina Next Seq 500 from SARS-CoV-infected primary human bronchial epithelium (NHBE) and mock-treated NHBE cells obtained from the Gene Expression Omnibus (GEO) (GSE147507), and the quality control (QC) was evaluated using the CLC Genomics Workbench 20.0 (Qiagen, United States) before the RNA-seq analysis using BioJupies web tool and iPathwayGuide for gene ontologies (GO), pathways, upstream regulator genes, small molecules, and natural products. Additionally, single-cell transcriptomics data (GSE163005) of meta clusters of immune cells from the cerebrospinal fluid (CSF), such as T-cells/natural killer cells (NK) (TcMeta), dendritic cells (DCMeta), and monocytes/granulocyte (monoMeta) cell types for comparison, namely, Neuro-COVID versus idiopathic intracranial hypertension (IIH), were analyzed using iPathwayGuide. L1000 fireworks display (L1000FWD) and L1000 characteristic direction signature search engine (L1000 CDS^2^) web tools were used to uncover the small molecules that could potentially reverse the COVID-19 and Neuro-COVID-associated gene signatures. We uncovered small molecules such as camptothecin, importazole, and withaferin A, which can potentially reverse COVID-19 associated gene signatures. In addition, withaferin A, trichostatin A, narciclasine, camptothecin, and JQ1 have the potential to reverse Neuro-COVID gene signatures. Furthermore, the gene set enrichment analysis (GSEA) preranked method and Metascape web tool were used to decipher and annotate the gene signatures that were potentially reversed by these small molecules. In conclusion, our study unravels a rapid approach for applying next-generation knowledge discovery (NGKD) platforms to discover small molecules with therapeutic potential against COVID-19 and its related disease pathologies.

## Introduction

Coronaviruses (CoVs) belong to the order Nidovirales, family Coronaviridae, and subfamily Coronavirinae, which can further be divided into four genera: alpha, beta, gamma, and delta CoVs. SARS CoV2 is the causative agent of coronavirus disease-19 (COVID-19), belongs to the genus beta-CoV, and can cause sickness varying from the common cold to more severe illnesses such as severe acute respiratory syndrome, gastrointestinal complications, sudden stroke, multiple organ failure, and mortality in some cases (Cui et al., 2019). SARS-CoV-2 infected more than 186 million people, resulting in the death of about 4 million people globally (Johns Hopkins COVID-19 Data Center on 10th July 2021) (Dong et al., 2020). SARS-CoV-2 has a positive-sense RNA genome encapsulated by a nucleocapsid. SARS-CoV-2 infects host cells through surface receptors, angiotensin-converting enzyme 2 (hACE2), and transmembrane protease serine-type 2 (TMPRSS2) (Hoffmann et al., 2020). An increase in the expression of ACE2, a tissue-protective mediator during lung damage, was found to be associated with interferon signaling in airway epithelial cells, and SARS-CoV-2 could exploit interferon-mediated stimulation of ACE2 to augment infection (Ziegler et al., 2020). The differential expression of genes that are necessary for SARS-CoV-2 interaction and subsequent host response determine susceptibility to COVID-19, disease progression, and recovery (Kasela et al., 2021).

RNA sequencing is a recently developed NGS methodology for whole transcriptome or single-cell transcriptomic approaches (Liu and Di, 2020). Single-cell RNA sequencing of COVID-19 infected bronchial epithelial cells and bronchioalveolar immune cells revealed important cellular and molecular processes implicated in COVID-19 infection at the single-cell level and provided information about the mechanisms of disease severity (Liu T. et al., 2020; Liao et al., 2020; Zhou et al., 2020). Notably, IL-17-associated signaling was significantly increased but not Th2-related inflammation following COVID-19 infection (Kasela et al., 2021)**.** A recent study showed that SARS-CoV-2 infection caused a twofold higher induction of interferon stimulation compared to SARS-CoV in Calu-3 human epithelial cells and subsequent induction of cytokines such as IL6 or IL-10 (Wyler et al., 2021). The interferon-induced genes IFIT2 and OAS2 were widely stimulated compared to interferon lambda (IFNL) and interferon-beta (IFNB). Besides, scRNA-seq data suggested that interferon regulatory factor (IRF) activity occurs before the induction of nuclear factor-κB (NF-κB) in SARS-CoV-2-infected cells (Wyler et al., 2021).

COVID-19 patients, especially those with greater disease severity, can develop neurological complications such as neuroinflammation, headache, and cerebrovascular disease called Neuro-COVID (Heming et al., 2021). Developing novel drug candidates and identifying suitable existing therapeutics for drug repurposing for COVID-19 and Neuro-COVID are critical for controlling this ongoing pandemic and reducing the enormous economic burden on health care systems and socioeconomic devastation of individuals, families, small to large businesses, and countries. Understanding COVID-19-associated gene signatures is essential for developing robust therapeutics for treating infected patients effectively and reducing infection rates and mortality. To address this important issue, the gene expression profiles of COVID-19 infection models can be used to identify potential therapeutic targets that could be targeted by known drugs. Here, we used RNA-seq datasets from the COVID-19 infection model of human bronchial epithelial cells (NHBE) and the scRNA-seq datasets of immune cells isolated from the cerebrospinal fluid (CSF) of Neuro-COVID patients, obtained from public repositories and analyzed using next-generation knowledge discovery (NGKD) platforms to understand disease-specific gene signatures and uncover drugs from synthetic and natural sources that can reverse these gene signatures for potential therapeutics.

## Materials and Methods

### Ethical Statement

This study was exempted from Institutional Review Board (IRB) approval since it did not involve any animal models or human subjects and was conducted using RNA-seq datasets retrieved from the Gene Expression Omnibus (GEO) (Barrett et al., 2013).

### Data Source

In the present study, the raw RNA-seq reads (Single-End) (*FASTQ format*) in quadruplicates derived using Illumina Next Seq 500 from SARS-CoV-infected and mock-treated NHBE cells were obtained from the GEO (*Accession No: GSE147507*) (Blanco-Melo, et al., 2020). Additionally, the single-cell transcriptomics data (*Accession No: GSE163005*) of immune cells isolated from the CSF of Neuro-COVID patients (Heming et al., 2021) were used for additional analysis using high-throughput knowledge discovery platforms. Heming et al. (2021) provided the entire dataset for the open-source interactive platform *cerebroApp* at http://covid.mheming.de/ (Hillje et al., 2020).

### COVID-19 RNA-seq Data: Quality Control (QC)

Raw RNA-seq reads (Single-End) (*FASTQ format*) in quadruplicates were evaluated for quality using the CLC Genomics Workbench 20.0 (Qiagen, United States) as described previously (Ewing and Green, 1998; Liu and Di, 2020).

### BioJupies Analysis of the RNA-seq Data

BioJupies is a freely available web-based application (http://biojupies.cloud) that has 14 RNA-seq analysis library plug-ins and provides the user with the automatic generation, storage, and deployment of Jupyter Notebooks containing RNA-seq data analyses (Torre et al., 2018). In BioJupies, the RNA-seq datasets were user-submitted, compressed in an HDF5 data package, and uploaded to Google Cloud. Raw counts were normalized to log10-Counts per million (log CPM) and the differentially expressed genes (DEGs) were derived between the control group and the experimental group using the limma R package (Ritchie et al., 2015). The Jupyter Notebooks created for each RNA-seq raw data analysis were permanently available through a URL and stored in the cloud. The notebooks consist of executable code of the whole pipeline, description of the methods, enrichment analysis, interactive data visualizations, differential expression, and so on (Torre et al., 2018).

Principal component analysis (PCA) was performed using the PCA function from the sklearn Python module by transforming the log CPM using the Z-score method. An interactive heatmap was generated using a clustergram (Fernandez et al., 2017). In the volcano plots, the log2 fold changes of the DEGs are shown on the *x*-axis and *p*-values were corrected using the Benjamini-Hochberg method, transformed (–log10), and presented on the *y*-axis (Benjamini and Hochberg, 1995; Benjamini and Yekutieli, 2001). In contrast, for the MA plot, average gene expression is shown on the x-axis; *p*-values were corrected using the Benjamini-Hochberg method (Benjamini and Hochberg, 1995; Benjamini and Yekutieli, 2001), transformed (–log10), and presented on the y-axis.

### *In Silico* Analysis of the RNA-seq Expression Data Using iPathwayGuide

The impact analysis method (IAM) (Draghici et al., 2007; Khatri et al., 2007; Tarca et al., 2009) was used to determine the significantly impacted gene signatures and pathways from the DEGs (log2FC cut-off 0.6, adjusted false discovery rate (FDR) *p*-value ≤ 0.05) obtained from the COVID-19 using BioJupies and the DEGs with log2FC (cut-off 0.3) and adjusted *p*-value ≤ 0.001 based on Bonferroni method in meta clusters of T-cells/natural killer cells (NK) (TcMeta), dendritic cells (DCMeta), and monocytes/granulocyte (monoMeta) cell types of the comparison, namely, Neuro-COVID versus idiopathic intracranial hypertension (IIH) for the Neuro-COVID infection models in the iPathwayGuide (Advaita Bioinformatics, United States). Here, the *p*-value calculated based on Fisher’s method was used to compute the pathway score method (Fisher, 1925). The *p*-value was further corrected based on multiple testing corrections for FDR and Bonferroni corrections (Bonferroni, 1935; Bonferroni, 1936). The gene interactions and pathways based on the DEGs were generated using the Kyoto Encyclopedia of Genes and Genomes (KEGG) database (Kanehisa and Goto, 2000; Kanehisa et al., 2002; Kanehisa et al., 2010; Kanehisa et al., 2012; Kanehisa et al., 2014). For each gene ontology (GO) term (Ashburner et al., 2000; Gene Ontology Consortium, 2001; Ashburner and Lewis, 2002; Gene Ontology Consortium, 2004), the number of DEGs annotated to the term was compared to that expected by chance. iPathwayGuide uses an overrepresentation approach to compute the statistical significance of observing at least a given number of DEGs (Draghici et al., 2003a; Draghici et al., 2003b; Draghici 2011). The hypergeometric distribution was used to compute the *p*-values in the iPathwayGuide analysis and corrected using FDR and Bonferroni for multiple comparisons (Draghici et al., 2003a; Draghici et al., 2003b; Draghici 2011). The prediction of upstream chemicals, drugs, and toxins (CDTs), either as present (or overly abundant) or absent (or insufficient), is based on two types of information: *1*) the enrichment of DEGs from the experiment and *2*) a network of interactions from the Advaita Knowledge Base (AKB v2012) (Draghici et al., 2003a; Draghici 2011). The analysis uses Fisher’s standard method to combine *p*-values into one test statistic (Fisher, 1925).

### L1000CDS2 and L1000FWD Queries

The L1000 characteristic direction signature search engine (L1000CDS2) analysis was performed by submitting the top 2000 DEGs to the L1000CDS2 signature search application programming interface (API) (Duan et al., 2016). Similarly, the L1000FWD analysis was performed by submitting the top 2000 DEGs to the L1000 Fire Works Display (L1000FWD) signature search API (Wang et al., 2018). Similarly, the DEGs obtained from TcMeta, DCMeta, and monoMeta cell types were compared; namely, Neuro-COVID versus IIH were subjected to both L1000CDS2 and L1000FWD analyses to identify drugs that reverse the gene signatures differentially regulated by COVID-19. An FDR (q-value) of 0.05 was considered statistically significant.

### Gene Set Enrichment Analysis (GSEA) Preranked

GSEA against a ranked list of genes was performed using the GSEA preranked method (Subramanian et al., 2005). The RNK-formatted files were created to the comparison of SARS-CoV-2-NHBE vs. Mock-NHBE, Neuro-COVID vs. IIH-TcMeta, Neuro-COVID vs. IIH-DCMeta, and Neuro-COVID vs. IIH-monoMeta, based on the ranking metric log2FC of the DEGs. Gene matrix files (GMTs) were created using the gene signatures (combined, up, and down) of withaferin A, importazole, camptothecin, trichostatin A, narciclasine, and JQ1 from the L1000FWD web tool (Wang et al., 2018). GSEA preranked was run by weighting each gene’s contribution to the enrichment score by the value of its ranking metric against GMT files using Java-based desktop application GSEA 4.1.0 (Broad Institute, United States) under default settings as described previously (Subramanian et al., 2005).

### Metascape Analysis of Gene Signatures Reversed by Small Molecules

The Metascape web tool (http://metascape.org) offers an easy and effective way to explore and understand gene lists derived from experimental data. The gene signatures reversed by small molecules identified in our study in COVID-19 and Neuro-COVID models were first automatically converted into Human Entrez Gene ID in Metascape. Then, all statistically enriched terms, accumulative hypergeometric *p*-values, and enrichment factors were calculated and used for filtering to obtain enrichment ontology clusters based on GO/KEGG terms, canonical pathways, and hallmark gene sets (Zhou et al., 2019).

## Results

Raw RNA-seq reads (Single-End) (*FASTQ format*) derived using Illumina Next Seq 500 from SARS-CoV-infected NHBE and mock-treated NHBE cells were obtained from the GEO and the QC was evaluated using the CLC Genomics Workbench 20.0, before the RNA-seq analysis using BioJupies web tool. iPathwayGuide analysis was performed to decipher the disease-specific signatures, pathways, and small molecules, either synthetic or derived from natural sources, to reverse disease-specific gene signatures. In addition, single-cell transcriptomic data of immune cells isolated from the CSF of Neuro-COVID-19 patients were further analyzed using iPathwayGuide, L1000CDS2, and L1000FWD analyses.

Hierarchically clustered heatmaps were generated using the Clustergrammer web tool to visualize and analyze high-dimensional RNA-seq data of SARS-CoV-infected NHBE cells and mock-treated NHBE cells (Supplementary Figure S1A). PCA was used to uncover global patterns in RNA-seq datasets analyzed and helped to understand the difference between COVID-19-infected and mock-treated NHBE cells (Supplementary Figure S1B). The volcano plot was generated using transformed gene fold changes using log2 and is shown on the *x*-axis (Supplementary Figure S1C). The MA plot was based on the average gene expression, which was calculated using the mean of the normalized gene expression values and shown on the *x*-axis (Supplementary Figure S1D).

### iPathwayGuide Analysis of DEGs From COVID-19 and Neuro-COVID Infection Models

In this experiment, 1,072 DEGs were identified from a total of 10,663 DEGs obtained from BioJupies analysis of the RNA-seq reads of the SARS-CoV-infected NHBE cells and mock-treated NHBE cells based on a *p*-value cut-off of 0.05 and a log2 fold change cut-off of 0.6. In contrast, DEGs with a logFC cut-off of 0.3 and adjusted *p*-value based on the Bonferroni method from clusters in TcMeta, DCMeta, and monoMeta of the comparison, namely, Neuro-COVID versus IIH, were also subjected to iPathwayGuide analysis separately, followed by comparative analyses. Subsequently, the DEGs were analyzed in the context of pathways obtained from the KEGG database (Kanehisa and Goto, 2000; Kanehisa et al., 2002), GO from the Gene Ontology Consortium database, a network of regulatory relationships from BioGRID: Biological General Repository for Interaction Datasets v4.0.189 (Szklarczyk et al., 2017), chemicals/drugs/toxicants from the Comparative Toxicogenomics Database (Davis et al., 2021), and diseases from the KEGG database. In summary, 22 pathways were found to be significantly impacted in SARS-CoV-2-infected NHBE cells compared to mock-treated NHBE cells. In addition, 503 GO terms, 18 miRNAs, 190 gene upstream regulators, 213 chemical upstream regulators, and 14 diseases were found to be significantly enriched before correction for multiple comparisons.

COVID-19 infection of NHBE cells triggers key immune-related pathways, such as cytokine-cytokine receptor interactions and viral protein interactions with cytokine receptors (Table 1). The top five upstream regulators, IL-17, TNF-alpha, STAT2, IRF9, and TLR4, were predicted to be activated (Table 2). The top identified biological processes, molecular functions, and cellular components for each pruning type are provided in Tables 3–5.

**TABLE 1:** Top identified pathways and their associated *p*-values are given in the table.

Pathway name	Pathway ID	*p*-value	*p*-value (FDR)	*p*-value (Bonferroni)
Cytokine-cytokine receptor interaction	04060	6.711e−8	2.0202e−5	2.020e−5
*Staphylococcus aureus* infection	05150	4.009e−7	6.034e−5	1.207e−4
Viral protein interaction with cytokine and cytokine receptor	04061	1.050e−7	1.053e−4	3.160e−4
Systemic lupus erythematosus	05322	3.414e−4	0.026	0.103
Herpes simplex virus 1 infection	05168	6.444e−4	0.039	0.194

The *p*-value corresponding to the pathway was calculated based on overrepresentation analysis.

**TABLE 2:** Top identified upstream regulators activated based on Bonferroni correction were listed in the table.

Upstream regulator (u)	DTA (u)	DT (u)	*p*-value	*p*-value (FDR)	*p*-value (Bonferroni)
IL17A	13	17	1.139e−6	0.001	0.001
TNF	23	28	1.857e−6	0.001	0.002
STAT2	9	9	5.589e−6	0.002	0.007
IRF9	8	8	2.248e−5	0.007	0.028
TLR4	8	8	8.493e−5	0.021	0.106

Table 2 indicates the number of differentially expressed (DE) targets supporting the hypothesis that each upstream regulator (u) is activated (DTA(u)), the total number of DE genes downstream of u (DT(u)), the combined raw p-value, and the corrected p-value for multiple comparisons.

**TABLE 3:** Top identified biological processes for each pruning type.

Pruning type: none				Pruning type: high specificity		Pruning type: smallest common denominator	
Go term	*p*-value	*p*-value (FDR)	*p*-value (Bonferroni)	Go term	*p*-value	Go term	*p*-value
Keratinization	6.600e−9	4.710e−5	4.710e−5	Cornification	1.713e−4	Keratinization	4.710e−5
Cornification	2.400e−8	8.563e−5	1.713e−4	Acute-phase response	0.033	Acute-phase response	0.100
Humoral immune response	2.900e−7	6.898e−4	0.002	Peptide cross-linking	0.209	Humoral immune response	0.167
DNA replication initiation	9.000e−6	0.002	0.064	Double-strand break repair *via* break-induced replication	0.371	Peptide cross-linking	0.371
Acute-phase response	9.300e−6	0.012	0.066	Antimicrobial humoral immune response mediated by antimicrobial peptide	0.371	Double-strand break repair *via* break-induced replication	0.371

**TABLE 4:** Top identified molecular functions for each pruning type.

Pruning type: none				Pruning type: high specificity		Pruning type: smallest common denominator	
Go term	*p*-value	*p*-value (FDR)	*p*-value (Bonferroni)	Go term	*p*-value	Go term	*p*-value
Cytokine activity	1.200e−7	1.691e−4	1.691e−4	Cytokine activity	0.061	Cytokine activity	1.691e−4
Receptor regulator activity	6.500e−7	4.579e−4	9.158e−4	Chemokine activity	0.061	Serine hydrolase activity	0.341
Signaling receptor activator activity	4.500e−6	0.002	0.006	DNA replication origin binding	0.341	DNA replication origin binding	0.341
Receptor-ligand activity	7.900e−6	0.003	0.011	2’-5’-Oligodenylate synthetase activity	0.341	2’-5’-Oligodenylate synthetase activity	0.341
Chemokine activity	6.000e−5	0.017	0.085	Structural constituent of skin epidermis	0.341	Structural constituent of skin epidermis	0.341

**TABLE 5:** Top identified cellular components for each pruning type.

Pruning type: none				Pruning type: high specificity		Pruning type: smallest common denominator	
Go term	*p*-value	*p*-value (FDR)	*p*-value (Bonferroni)	Go term	*p*-value	Go term	*p*-value
Cornified envelope	3.600e−5	0.024	0.033	Cornified envelope	0.033	Cornified envelope	0.024
Intermediate filament	5.200e−5	0.024	0.048	Intermediate filament	0.279	Intermediate filament	0.024
DNA packaging complex	2.700e−4	0.082	0.247	Blood microparticle	0.485	DNA packaging complex	0.082
Intermediate filament cytoskeleton	0.001	0.209	0.924	MCM complex	0.492	Blood microparticle	0.364
Extracellular matrix	0.001	0.209	1.000	Nucleosome	0.492	MCM complex	0.470

**FIGURE 1:**
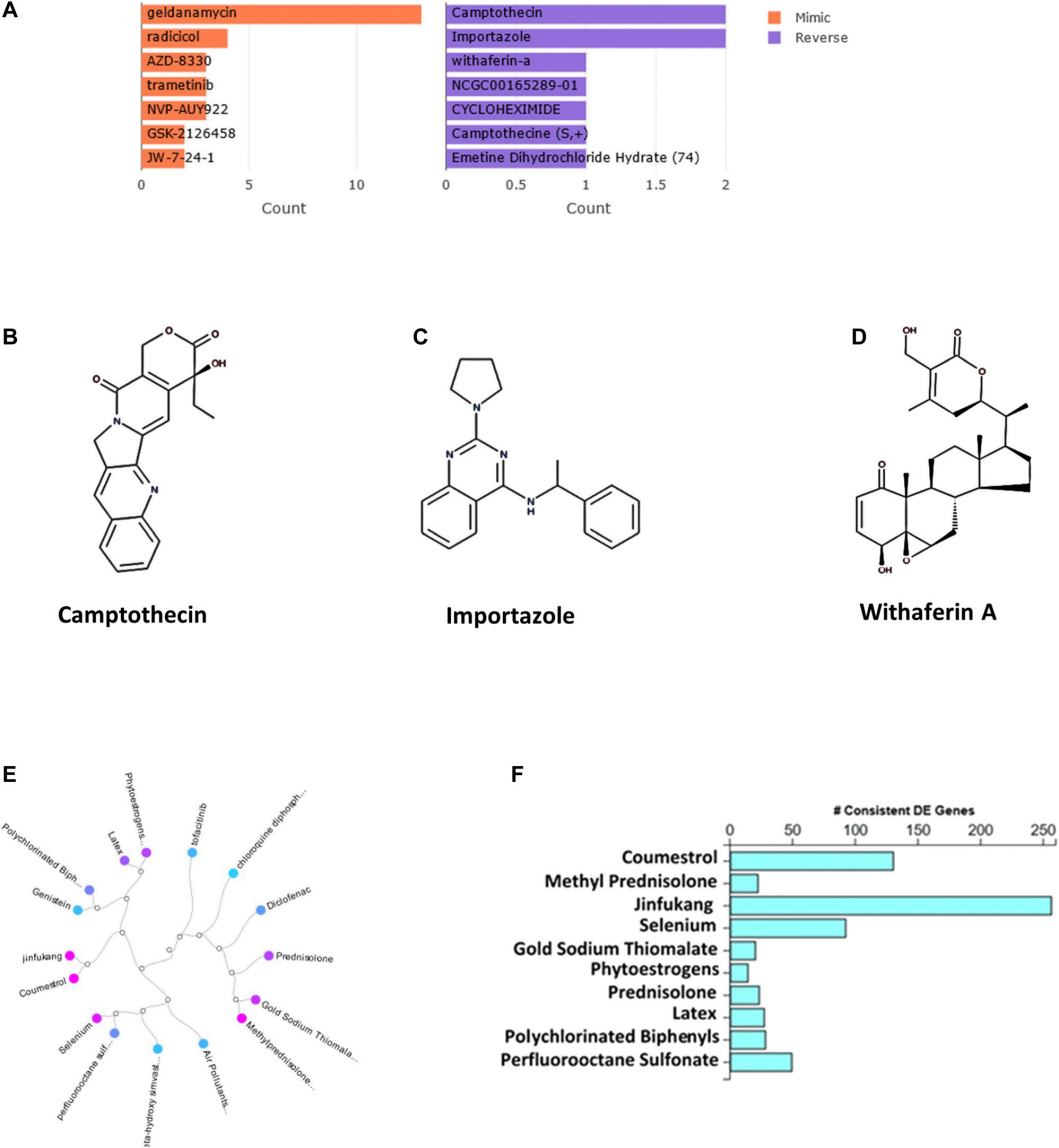
**(A)** The bar chart displaying the top five small molecules identified by the L1000CDS2 query. The left panel displays the small molecules which mimic the observed gene expression signature, while the right panel displays the small molecules which reverse it. **(B**–**D)** The two-dimensional structures of camptothecin, importazole, and withaferin A. **(E)** Dendrogram show the drugs and natural products that reverse the COVID-19 associated molecular signatures and **(F)** bar graph shows the drugs and natural products that potentially reverse COVID-19 associated molecular signatures based on iPathwayGuide analysis.

The bar chart (Figure 1A) shows the top small molecules identified by the L1000CDS2 query using the DEGs identified from SARS-CoV-2-NHBE. The left panel shows small molecules such as geldanamycin, radicicol, AZD8330, trametinib, NVP-AYU922, GSK2126458, and JW-7-24-1, which mimic the observed gene expression signature; the right panel displays small molecules such as camptothecin (Figure 1B), importazole (Figure 1C), and withaferin A (Figure 1D). The upstream regulator drugs and natural products that reverse the molecular signatures based on iPathwayGuide analysis are shown as a dendrogram (Figure 1E). The top five upstream drugs, natural products, and chemicals predicted as absent (or insufficient) based on iPathwayGuide analysis were coumestrol, methylprednisolone, JinFuKang (JFK), selenium, and gold sodium thiomalate (Figure 1F). However, withaferin A was found to reverse the COVID-19-induced molecular signatures in both L1000CDS2 and L1000FWD analyses, along with other small-molecule drugs (Table 6) in the SARS-CoV-2-infected NHBE cells.

**TABLE 6:** Natural products and drugs with opposite molecular signatures based on L1000FWD web-based tool for querying gene expression signatures (SARS-CoV-2-NHBE vs. Mock-NHBE) against signatures created from human cell lines treated with over 20,000 small molecules and drugs for the LINCS project.

Signature ID	Drug or natural product	Similarity SCORE	*p*-value	q-value	Z-score	Combined score
CPC006_PC3_6H:BRD-A36630025-001-02-6:0.35	SN-38	−0.0598	1.04e−11	2.02e−08	1.79	−19.61
CPC011_A549_24H:BRD-K97514127-045-02-0:10	Vinorelbine	−0.0598	2.32e−11	3.82e−08	1.72	−18.26
CPC015_MCF7_24H:BRD-K52075715-001-03-4:10	Oxibendazole	−0.0573	2.91e−10	3.37e−07	1.67	−15.88
CPC019_HT29_6H:BRD-K67870070-001-01-4:10	SA-247615	−0.0560	7.96e−11	1.22e−07	1.69	−17.05
ERG005_VCAP_6H:BRD-K88378636-001-02-8:20	Withaferin A	−0.0547	1.28e−09	1.19e−06	1.65	−14.71
CPC012_MCF7_24H:BRD-K69496360-001-01-5:10	BRD-K69496360	−0.0522	1.03e−08	5.72e−06	1.73	−13.81
CPC015_MCF7_24H:BRD-K47869605-001-18-9:10	Podophyllotoxin	−0.0509	1.78e−08	8.35e−06	1.67	−12.97
CPC001_HCC515_24H:BRD-K82823804-001-01-7:10	SA-792987	−0.0509	3.61e−08	1.44e−05	1.81	−13.45
MUC.CP003_MCF7_24H:BRD-K02407574-001-04-8:3.3333	Parbendazole	−0.0496	8.46e−08	2.85e−05	1.62	−11.45
CPC002_PC3_6H:BRD-K06926592-001-01-7:10	Tretinoin	−0.0496	7.24e−08	2.54e−05	1.79	−12.80

**FIGURE 2:**
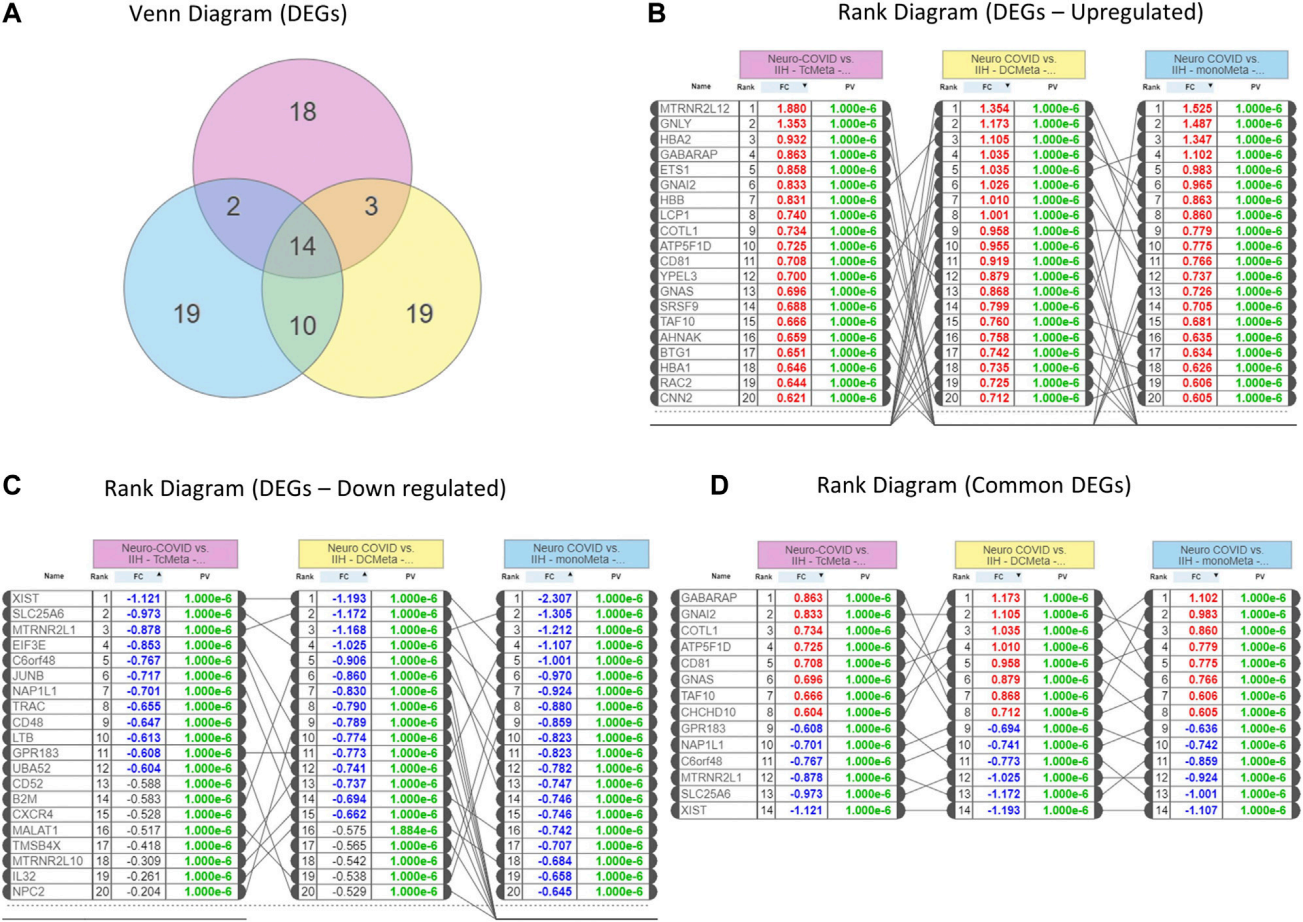
Differentially expressed genes (DEGs) in Neuro-COVID vs. IIH comparison based on iPathwayGuide analysis. **(A)** Venn diagram shows the DEGs between TcMeta, DCMeta, and monoMeta. **(B)** The rank diagram shows the upregulated DEGs in TcMeta, DCMeta, and monoMeta. **(C)** The rank diagram shows the downregulated DEGs in TcMeta, DCMeta, and monoMeta. **(D)** The rank diagram shows the common DEGs between TcMeta, DCMeta, and monoMeta.

We identified 14 genes that were commonly expressed between Neuro-COVID and IIH (TcMeta, DCMeta, and monoMeta), as depicted in the Venn diagram (Figure 2A). The upregulated genes (Figure 2B), downregulated genes (Figure 2C), and the common genes between the meta clusters of immune cells in Neuro-COVID were presented as rank diagrams based on log2FC values. The genes GABARAP, GNAI2, COTL1, ATP5F1D, CD81, GNAS, TAF10, and CHCHD10 were significantly upregulated, and genes such as XIST, SLC25A6, MTRNR2L1, C6orf48, NAP1L1, and GPR183 were significantly downregulated in the meta clusters of immune cells in Neuro-COVID (Figure 2D).

**FIGURE 3:**
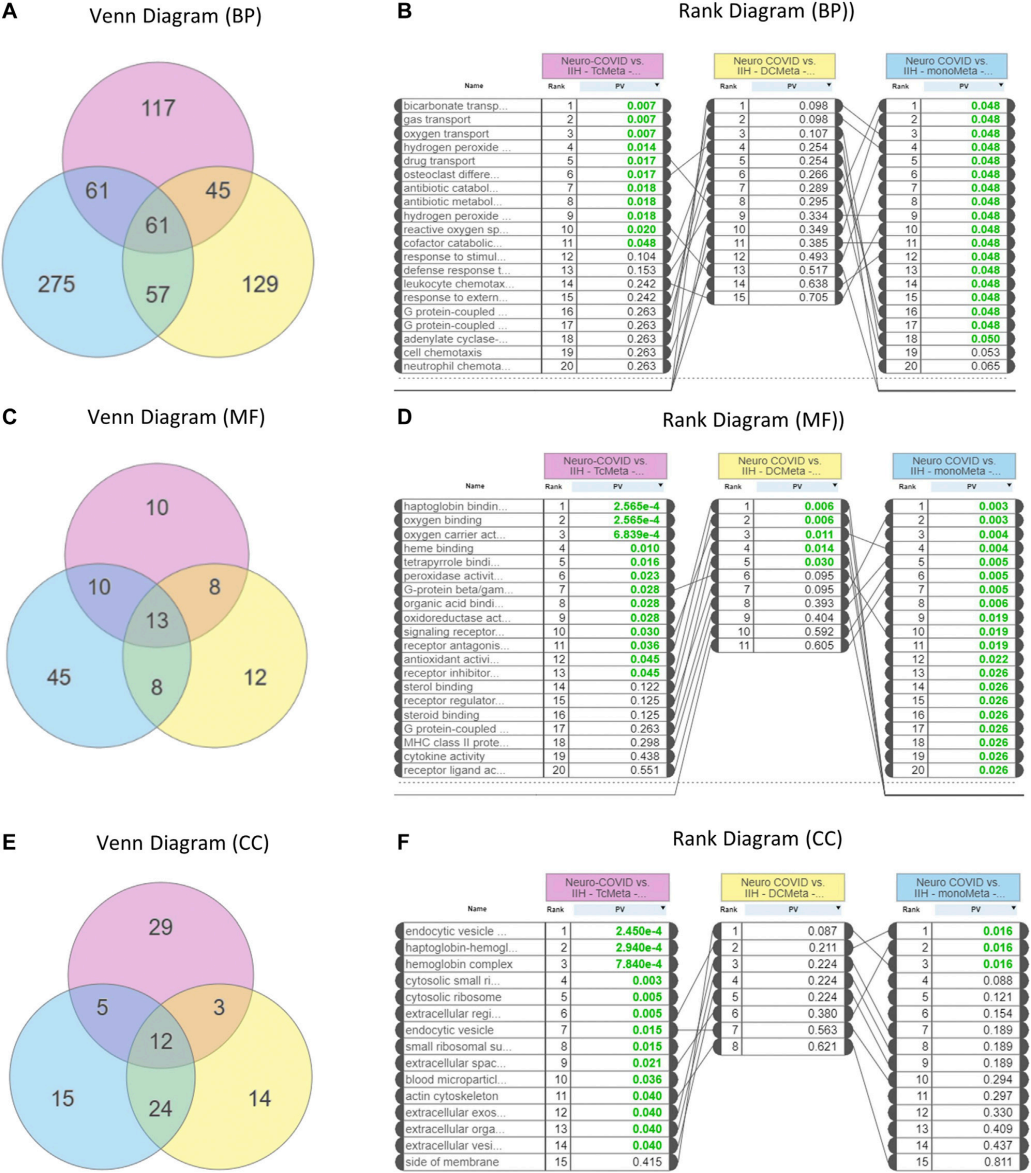
Enrichment of gene ontologies (GO) such as biological process (BP), molecular function (MF), and cellular component (CC) in Neuro-COVID vs. IIH comparison based on iPathwayGuide analysis. **(A)** Venn and **(B)** rank diagrams show the BP enriched between TcMeta, DCMeta, and monoMeta. **(C)** Venn and **(D)** rank diagrams show the MF enriched between TcMeta, DCMeta, and monoMeta. **(E)** Venn and **(F)** rank diagrams show the CC enriched between TcMeta, DCMeta, and monoMeta.

GO analysis showed that 61 biological processes (Figure 3A), 13 molecular functions (Figure 3C), and 12 cellular components (Figure 3E) were commonly enriched in the meta clusters of immune cells in Neuro-COVID. The top five biological processes enriched were bicarbonate transport, gas transport, oxygen transport, hydrogen peroxide, and drug transport (Figure 3B), the top five molecular functions enriched were haptoglobin binding, oxygen binding, oxygen carrier activity, heme binding, and tetrapyrrole binding (Figure 3D), and the top five cellular components enriched were endocytic vesicle, haptoglobin-hemoglobin complex, hemoglobin complex, cytosolic small ribosomes, and cytosolic ribosomes (Figure 3F). The top five differentially expressed pathways identified based on iPathwayGuide analysis of immune cell meta clusters from Neuro-COVID patients were malaria, African trypanosomiasis, cocaine addiction, Parkinson’s disease, and leukocyte transendothelial migration. The differentially regulated pathways in the meta clusters of immune cells from patients with Neuro-COVID are provided in Supplementary Table S1. The upstream genes activated in TcMeta, DCMeta, and monoMeta clusters are listed in Supplementary Table S2.

### L1000FWD and L1000CDS2 Analyses

**FIGURE 4:**
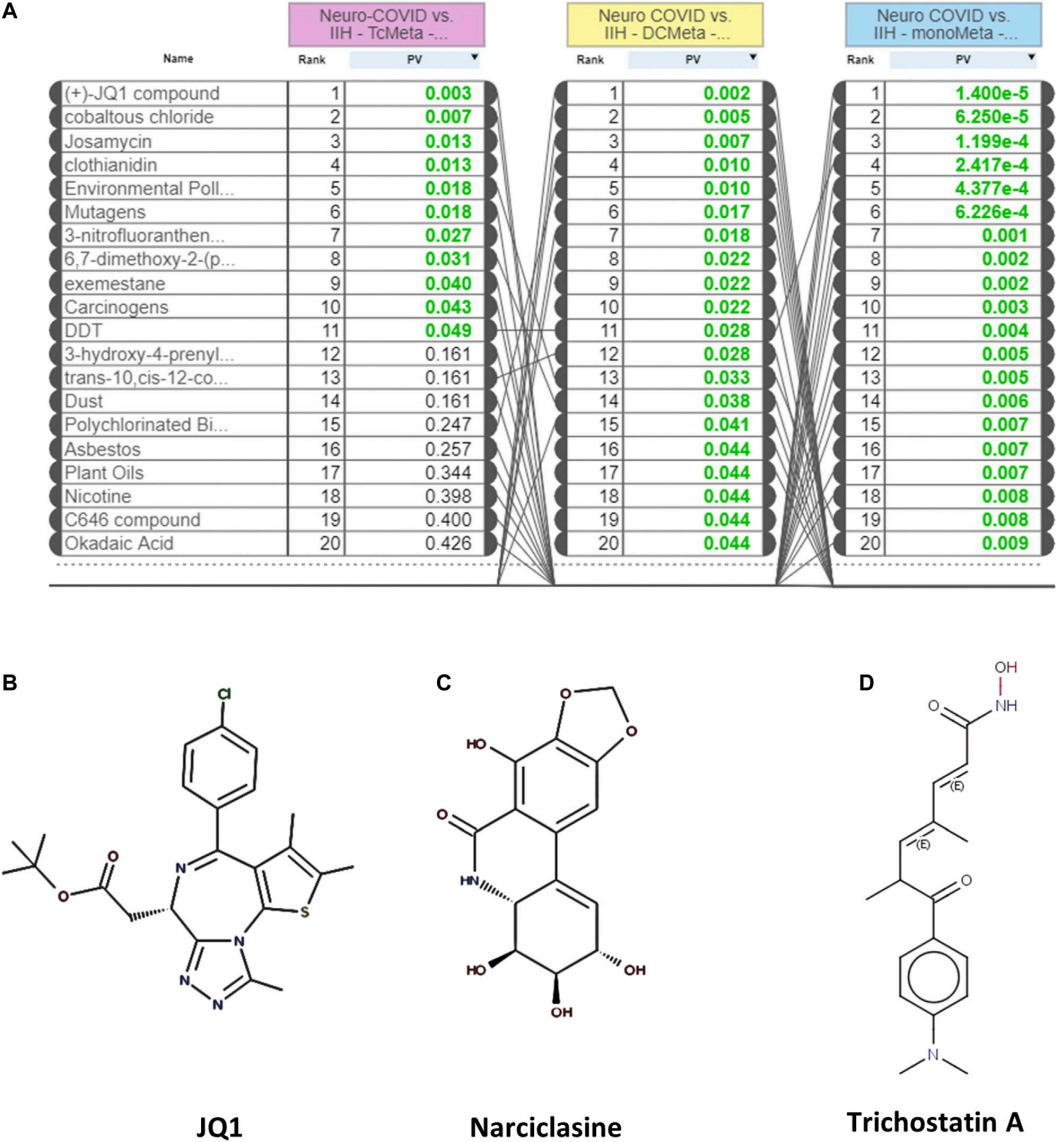
**(A)** The rank diagram based on the *in silico* prediction of insufficient signaling of drugs and natural products in TcMeta, DCMeta, and monoMeta using iPathwayGuide analysis. **(B**–**D)** The two-dimensional structures of JQ1, narciclasine, and trichostatin A.

The L1000FWD analysis of DEGs of meta clusters of Tc, DC, and Mono of Neuro-COVID compared to IIH revealed that withaferin A was the top molecule capable of reversing the COVID-19 induced gene signatures (Tables 7–9). Furthermore, the rank diagram (Figure 4A) showed that JQ1 was the top drug based on the *in silico* prediction of insufficient signaling of drugs, natural products, and chemicals in the meta clusters of Tc, DC, and Mono of Neuro-COVID compared to IIH using iPathwayGuide (Figure 4B). The L100CDS2 analysis of DEGs of meta clusters of Tc, DC, and Mono of Neuro-COVID compared to IIH revealed that narciclasine (Figure 4C) and trichostatin A (Figure 4D) were some of the top molecules potentially reversing the Neuro-COVID gene signatures (Tables 10–12).

**TABLE 7:** Top small molecules with opposite molecular signatures based on L1000FWD web-based tool for querying gene expression signatures (Neuro-COVID vs. IIH-Tc Meta) against signatures created from human cell lines treated with over 20,000 small molecules and drugs for the LINCS project.

Signature ID	Drug or natural product	Similarity score	*p*-value	q-value	Z-score	Combined score
ERG005_VCAP_6H:BRD-K88378636-001-02-8:20	Withaferin A	−0.0492	3.55e−16	1.52e−11	1.65	−25.55
CPC012_VCAP_24H:BRD-A59985574-003-01-9:10	Topotecan	−0.0303	6.34e−05	4.35e−01	1.75	−7.36
CPC012_VCAP_24H:BRD-K62459624-001-08-7:10	BRD-K62459624	−0.0293	1.66e−04	8.86e−01	1.77	−6.69
CPC006_HCC515_6H:BRD-K16406336-311-01-2:10	Methylene-blue	−0.0303	8.54e−04	1.00e+00	1.77	−5.43
CPC011_PC3_6H:BRD-K04548931-003-11-6:10	Pidorubicine	−0.0278	1.04e−03	1.00e+00	1.77	−5.27
NMH002_NPC_24H:BRD-K32610195-001-14-9:10	Androstenedione	−0.0269	1.58e−03	1.00e+00	1.63	−4.57
CPC012_VCAP_24H:BRD-K56196992-001-01-2:10	BRD-K56196992	−0.0269	9.60e−03	1.00e+00	1.72	−3.47
CPC015_NPC_24H:BRD-K14920963-304-01-9:10	Erythrosine	−0.0269	1.51e−03	1.00e+00	1.75	−4.93
CPC013_SKB_24H:BRD-K16798053-001-01-0:10	ST-4029573	−0.0269	4.94e−04	1.00e+00	1.76	−5.82
CPC004_PC3_6H:BRD-A41519720-001-03-0:10	Ezetimibe	−0.0264	1.21e−02	1.00e+00	1.78	−3.42

**TABLE 8:** Top small molecules with opposite molecular signatures based on L1000FWD web-based tool for querying gene expression signatures (Neuro-COVID vs. IIH-DC Meta) against signatures created from human cell lines treated with over 20,000 small molecules and drugs for the LINCS project.

Signature ID	Drug or natural product	Similarity score	*p*-value	q-value	Z-score	Combined score
ERG005_VCAP_6H:BRD-K88378636-001-02-8:20	Withaferin A	−0.0587	2.84e−19	1.21e−14	1.65	−30.67
CPC015_NPC_24H:BRD-K14920963-304-01-9:10	Erythrosine	−0.0304	2.85e−04	7.64e−01	1.75	−6.19
CPC006_HCC515_6H:BRD-K16406336-311-01-2:10	Methylene-blue	−0.0330	3.94e−04	8.88e−01	1.77	−6.02
CPC012_HCC515_6H:BRD-K56653679-001-01-2:10	MD-041	−0.0317	4.22e−04	9.03e−01	1.72	−5.81
CVD001_HUH7_6H:BRD-K81142122-001-14-1:10	STK-249718	−0.0323	3.91e−04	8.88e-01	1.62	−5.52
CPC004_HT29_6H:BRD-K77830450-001-02-4:10	Forskolin	−0.0264	1.31e−03	1.00e+00	1.90	−5.47
CPC013_SKB_24H:BRD-K16798053-001-01-0:10	ST-4029573	−0.0277	9.86e−04	1.00e+00	1.76	−5.29
CPC014_HA1E_6H:BRD-U66370498-000-01-0:10	Androstanol	−0.0264	1.43e−03	1.00e+00	1.77	−5.03
CPC005_A375_24H:BRD-A78360835-001-01-1:10	Cercosporin	−0.0290	2.06e−03	1.00e+00	1.82	−4.90
CPC014_HCC515_6H:BRD-A80960055-001-01-7:10	Celastrol	−0.0290	2.78e−03	1.00e+00	1.72	−4.40

**TABLE 9:** Top small molecules with opposite molecular signatures based on L1000FWD web-based tool for querying gene expression signatures (Neuro-COVID vs. IIH-monoMeta) against signatures created from human cell lines treated with over 20,000 small molecules and drugs for the LINCS project.

Signature ID	Drug or natural product	Similarity score	*p*-value	q-value	Z-score	combined score
ERG005_VCAP_6H:BRD-K88378636-001-02-8:20	Withaferin A	−0.0533	2.68e−13	1.06e−08	1.65	−20.79
CPC006_SNUC5_6H:BRD-A19633847-050-20-6:10	Perhexiline	−0.0414	4.51e−08	3.85e−04	1.80	−13.23
CPC013_SKB_24H:BRD-K16798053-001-01-0:10	ST-4029573	−0.0382	2.01e−07	8.62e−04	1.76	−11.79
CPC017_SKB_24H:BRD-A20968261-001-01-3:10	WAY-213613	−0.0414	2.81e−07	1.00e−03	1.68	−11.01
CPC006_CORL23_6H:BRD-A04706586-236-01-7:10	Bucladesine	−0.0358	2.08e−06	4.95e−03	1.84	−10.48
CPC007_A375_24H:BRD-K03067624-003-19-3:10	Emetine	−0.0398	1.42e−06	3.80e−03	1.78	−10.42
CPC007_A375_6H:BRD-K03067624-003-19-3:10	emetine	−0.0374	3.86e−06	8.48e−03	1.81	−9.79
CPC012_MCF7_6H:BRD-K41652870-001-01-9:10	BRD-K41652870	−0.0366	7.70e−06	1.32e−02	1.75	−8.97
CPC005_A375_24H:BRD-A78360835-001-01-1:10	Cercosporin	−0.0358	2.49e−05	2.74e−02	1.83	−8.40
CPC006_PL21_6H:BRD-K78659596-001-01-3:10	MLN-2238	−0.0334	3.27e−05	3.33e−02	1.85	−8.28

**TABLE 10:** Top small molecules identified by the L1000CDS2 query that reverse the Neuro-COVID vs. IIH-TcMeta gene signature.

Rank	Overlap	Perturbation	Cell line	Dose	Time (h)
1	0.0358	T5212475	VCAP	10.0 µm	24.0
2	0.0353	BRD-K56411643	VCAP	10.0 µm	24.0
3	0.0278	F3055	A375	10.0 µm	6.0
4	0.0254	Narciclasine	HA1E	10.0 µm	24.0
5	0.0249	Ro 31-8220 mesylate	HCC515	10.0 µm	24.0
6	0.0244	Erythrosine sodium	HA1E	10.0 µm	24.0
7	0.0244	Parthenolide	A375	20.0 µm	24.0
8	0.0239	Teniposide	A375	1.25 µm	24.0
9	0.0239	Daunorubicin	A549	10.0 µm	6.0
10	0.0239	EI-293	PC3	10.0 µm	6.0

**TABLE 11:** Top small molecules identified by the L1000CDS2 query that reverse the Neuro-COVID vs. IIH-DCMeta gene signature.

Rank	Overlap	Perturbation	Cell line	Dose	Time (h)
1	0.0337	Trichostatin A	A375	10.0 µm	24.0
2	0.0304	Narciclasine	HA1E	10.0 µm	24.0
3	0.0297	Camptothecin (S,+)	PC3	10.0 µm	24.0
4	0.0277	Erythrosine sodium	HA1E	10.0 µm	24.0
5	0.0277	Vorinostat	A375	11.1 µm	24.0
6	0.0271	F3055	A375	10.0 µm	6.0
7	0.0264	Parthenolide	A375	20.0 µm	24.0
8	0.0257	Curcumin	MCF7	48.0 µm	24.0
9	0.0257	BRD-K56411643	VCAP	10.0 µm	24.0
10	0.0257	Celastrol	HME1	10 µm	3

**TABLE 12:** Top small molecules identified by the L1000CDS2 query that reverse the Neuro-COVID vs. IIH-monoMeta gene signature.

Rank	Overlap	Perturbation	Cell line	Dose	Time (h)
1	0.0398	Trichostatin A	A375	10.0 µm	24.0
2	0.0390	Narciclasine	A375	10.0 µm	24.0
3	0.0366	Parthenolide	A375	20.0 µm	24.0
4	0.0350	HY-10518	VCAP	10.0 µm	24.0
5	0.0334	Vorinostat	A375	11.1 µm	24.0
6	0.0326	Teniposide	A375	1.25 µm	24.0
7	0.0326	BRD-K56411643	VCAP	10.0 µm	24.0
8	0.0326	BL-081	VCAP	10.0 µm	24.0
9	0.0326	Camptothecin (S,+)	PC3	10.0 µm	24.0
10	0.0310	Erythrosine sodium	HA1E	10.0 µm	24.0

### GSEA Preranked and Metascape Analyses

To obtain the specific gene signatures potentially reversed by camptothecin, importazole, and withaferin A, GSEA preranked analysis was performed using ranked DEGs from SARS-CoV-2-NHBE vs. Mock-NHBE comparison against gene signatures differentially regulated by these small molecules derived from the L1000FWD web tool. The gene signature (Signature ID: CPC002_PC3_24H: BRD-A30437061:10.0) downregulated by camptothecin was positively enriched (normalized enrichment score (NES) = 1.32**,** and q-value = 0.065) and the upregulated genes were negatively enriched (NES = −1.12 and q-value = 0.27) in the SARS CoV2-NHBE cells (Supplementary Figure S2A). The gene signature (Signature ID: CPC006_A375_24H: BRD-A02481876:60.0) downregulated by importazole was positively enriched (NES = 1.31 and q-value = 0.036) (Supplementary Figure S2B) and the gene signature (Signature ID: CPC014_VCAP_6H: BRD-A52193669:10.0) downregulated by withaferin A was significantly enriched (NES = 1.21 and q-value = 0)**,** and the upregulated genes were negatively enriched (NES = −1.21 and q-value = 0.14) in the SARS CoV2-NHBE cells (Supplementary Figure S2C).

**FIGURE 5:**
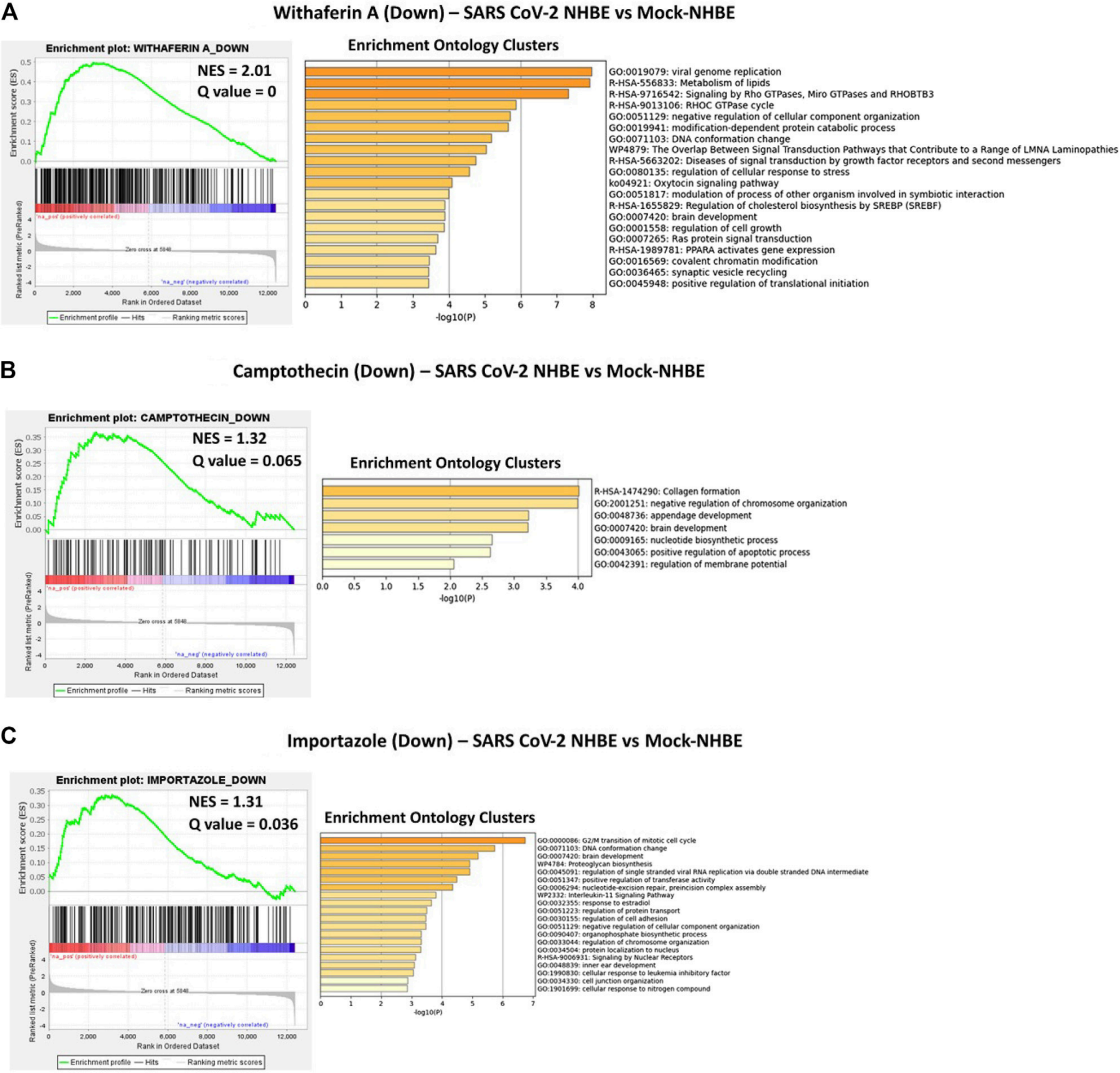
GSEA preranked analysis was performed to decipher the potential gene signatures downregulated by camptothecin, importazole, and withaferin A using the RNK file generated from DEGs of SARS CoV2-NHBE vs. Mock-NHBE comparison. The Metascape analyses to decipher the gene ontology clusters based on the **(A)** enrichment of gene signature (Signature ID: CPC014_VCAP_6H: BRD-A52193669:10.0) downregulated by withaferin A **(B)** enrichment of gene signature (Signature ID: CPC002_PC3_24H: BRD-A30437061:10.0) downregulated by camptothecin, and **(C)** enrichment of gene signature (Signature ID: CPC006_A375_24H: BRD-A02481876:60.0) downregulated by importazole in SARS CoV2-NHBE cells.

Camptothecin potentially reversed 28 genes that were positively enriched in SARS-CoV-2 in NHBE cells, and the top 10 genes were COL6A2, CSE1L, TMEM135, PPA2, MNAT1, BNIP3L, DLGAP5, TMEM47, ARHGAP29, and OLA1. In contrast, 11 genes upregulated by CPT were negatively enriched in SARS-CoV-2- NHBE cells, including RSAD2, CD74, HSPA2, SDC3, ZDHHC11, NEU1, S100A8, ISG15, MAFB, TSPAN7, and PEG3. Importazole potentially reversed 66 genes that were positively enriched in SARS-CoV-2-NHBE cells, and the top 10 genes were CDH19, CD58, TFF3, SNX10, SMC4, TMEM135, MNAT1, PBK, and TFPI. Withaferin A potentially reversed 134 genes that were positively enriched in SARS-CoV-2-NHBE cells, and the top 10 genes were NUDT4, CCNG1, ASPM, NLGN4X, USP1, SERP1, DIAPH2, PLEKHF2, XPO1, SUB1, SMC4, and HSPA6. In contrast, 23 genes upregulated by withaferin A were negatively enriched in SARS-CoV-2- NHBE cells and the top 10 genes were MT1F, CBR3, RAB20, SLC22A18, SLC37A4, EIF4EBP1, IRX5, S100A8, COL1A1, and ABHD14A. In addition, the gene signatures enriched in SARS-CoV-2-NHBE cells that were potentially reversed by withaferin A, camptothecin, and importazole were analyzed using Metascape to identify the enrichment ontology clusters based on GO/KEGG terms, canonical pathways, and hallmark gene sets (Figure 5). The genes enriched in GSEA preranked analysis of SARS CoV2-NHBE vs. Mock-NHBE against the gene signatures of camptothecin, importazole, and withaferin A are provided in Supplementary Datasheet S1.

Similarly, the GSEA preranked analysis was performed using ranked DEGs from Neuro-COVID vs. IIH-TcMeta, Neuro-COVID vs. IIH-DCMeta, and Neuro-COVID vs. IIH_monoMeta comparisons against gene signatures differentially regulated by withaferin A, camptothecin, trichostatin A, narciclasine, and JQ1 small molecules. The gene signature (Signature ID: CPC014_VCAP_6H: BRD-A52193669:10.0) upregulated by withaferin A was positively enriched (NES = 1.62, q = 0.028) in TcMeta, DCMeta (NES = 1.23, q-value <= 0.24)**,** and monoMeta (NES = 1.50, q-value = 0.06). However, the downregulated genes of withaferin A were moderately enriched in TcMeta and DCMeta (Supplementary Figure S3). The gene signature (Signature ID: CPC002_PC3_24H: BRD-A30437061:10.0) downregulated by camptothecin was significantly enriched (NES = 1.52, q = 0.051) in TcMeta and moderately enriched in DCMeta and monoMeta (Supplementary Figure S4).

The gene signature (Signature ID: CPC012_A375_6H: BRD-K68202742:10.0) downregulated by trichostatin A was moderately enriched (NES = 1.34 and q = 0.11) in TcMeta, DCMeta (NES = 0.92 and q-value = 0.59), and monoMeta (NES = 1.2 and q = 0.20). The upregulated genes of trichostatin A were negatively enriched in DCMeta (NES = −1.39 and q-value = 0.085) and monoMeta (NES = −1.47 and q-value = 0.10) (Supplementary Figure S5). The gene signature (Signature ID: CPC006_HA1E_24H: BRD-K06792661:10.0) upregulated by narciclasine was negatively enriched in TcMeta (NES = −1.97 and q-value = 0), DCMeta (NES = −1.65 and q-value = 0), and monoMeta (NES = −2.77 and q-value = 0). The differentially regulated genes of narciclasine were negatively enriched in monoMeta (NES = −2.01 and q-value = 0). However, the downregulated genes of narciclasine were moderately enriched in TcMeta (NES = 1.22 and q-value = 0.19) and DCMeta (NES = 1.32 and q = 0.15) (Supplementary Figure S6). The gene signature (Signature ID: LJP008_A549_24H: BRD-K54606188:10) downregulated by JQ1 was negatively enriched in the DCMeta (NES = −0.68 and q-value = 0.98) and Neuro-COVID vs. IIH-monoMeta (NES = −1.40 and q = 0.14) groups. The upregulated genes of JQ1 were moderately enriched in all three meta clusters of immune cells in Neuro-COVID (Supplementary Figure S7).

**FIGURE 6:**
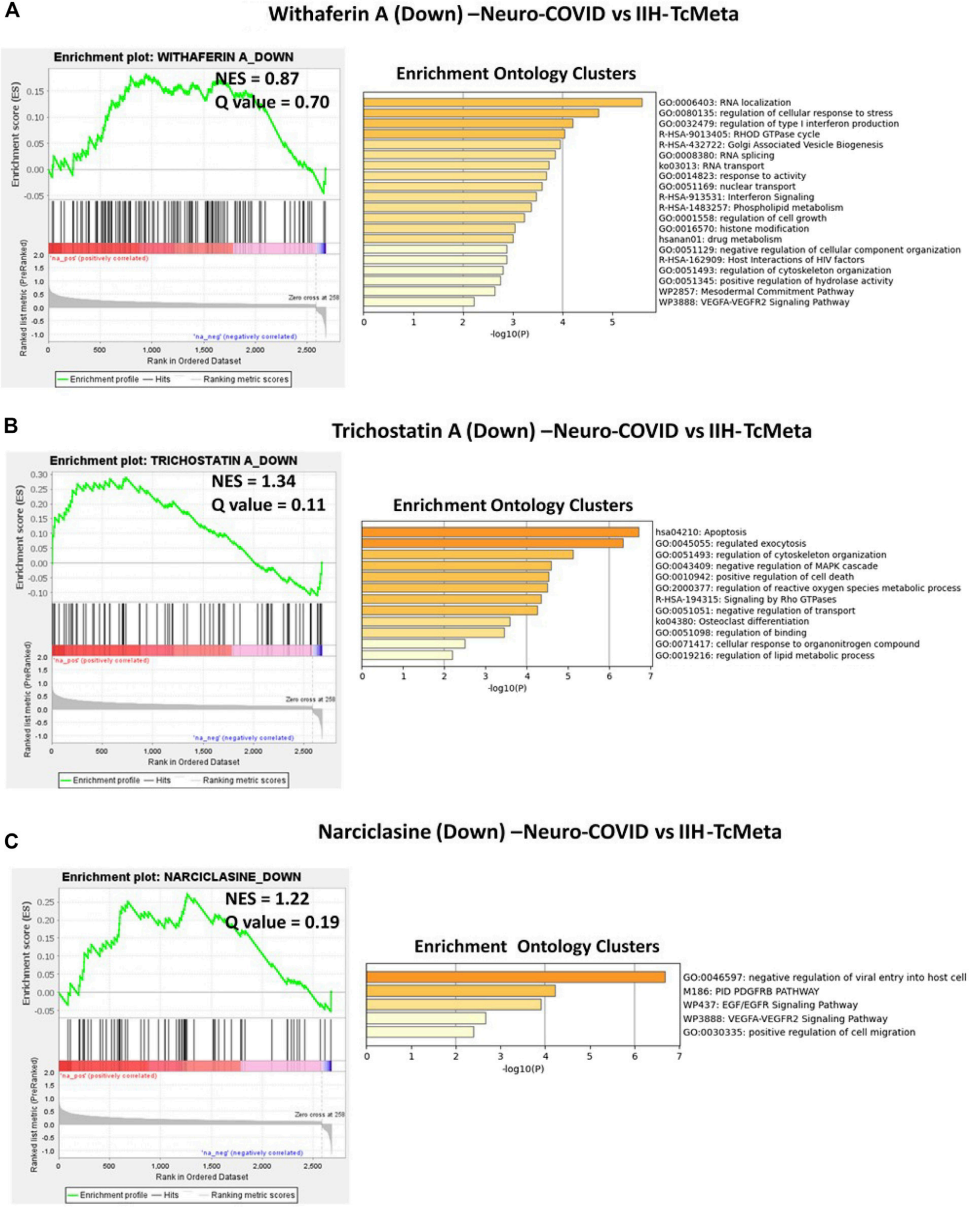
GSEA preranked analysis was performed to decipher the potential gene signatures downregulated by withaferin A, trichostatin A, and narciclasine using the RNK file generated from DEGs of Neuro-COVID vs. IIH (TcMeta) comparison. The Metascape analyses to decipher the GO clusters based on the **(A)** enrichment of gene signature (Signature ID: CPC014_VCAP_6H: BRD-A52193669:10.0) downregulated by withaferin A, **(B)** enrichment of gene signature (Signature ID: CPC012_A375_6H: BRD-K68202742:10.0) downregulated by trichostatin A, and **(C)** enrichment of gene signature (Signature ID: CPC006_HA1E_24H: BRD-K06792661:10.0) downregulated by narciclasine in Neuro-COVID vs. IIH-TcMeta.

The gene signatures upregulated by withaferin A (GNLY CST7 PPIB TSPO BCL2 S100A10 GSTP1), (S100A10, TSPO, PPIB, HLA-DQB1, BCL2, GSTP1, EDF1, and FLOT1), and (S100A9, S100A8, TSPO, and HOMER3) were positively enriched in TcMeta, DCMeta, and monoMeta clusters in Neuro-COVID. Camptothecin potentially reversed seven genes that were positively enriched in TcMeta, including AHNAK, MBNL1, LGALS1, HNRNPA2B1, S100A10, TGFBR2, and CAPN2. Trichostatin A potentially reverses five genes that were positively enriched in DCMeta, such as RGS2. IL32, ZFP36, SRGN, and STAB1, as well as nine genes that were positively enriched in monoMeta, such as SRGN, RGS2, IL32, ZFP36, JUNB, SAT1, PADI2, ALOX5AP, and IL2RG. The upregulated gene signatures of narciclasine (H3F3B, ZFP36, RGS2, and XIST) and (XIST, CREM, ZFP36, JUNB, NR4A2, FOS, EGR1, EVI2A, SAT1, EGR2, IER2, NR4A1, and KDM5A) were negatively enriched in DCMeta and monoMeta, respectively. JQ1 potentially increased six genes that were negatively enriched in DCMeta, such as H3F3B, AP2A2, PIGF, SOS1, TRIO, FHL3, H3F3B, MBNL2, TRIO, AP2A2, PSIP1, and ARHGEF6 in monoMeta. In addition, the gene signatures enriched in Neuro-COVID vs. IIH (TcMeta, DCMeta, and monoMeta) that are potentially reversed by withaferin A, camptothecin, trichostatin A, narciclasine, and JQ1 were analyzed using Metascape to find the enrichment ontology clusters based on GO/KEGG terms, canonical pathways, and hallmark gene sets. The enrichment ontology clusters derived for the gene signatures reversed by withaferin A, trichostatin A, and narciclasine in Neuro-COVID vs. IIH-TcMeta are shown in Figure 6. The genes enriched in GSEA preranked analysis of Neuro-COVID vs. IIH comparison against the gene signatures of withaferin A, camptothecin, trichostatin A, narciclasine, and JQ1 are provided in Supplementary Datasheet S2.

## Discussion

COVID-19 caused by SARS-CoV-2 infection remains an ongoing pandemic (Huang C. et al., 2020; Liu J. et al., 2020; Novel Coronavirus Pneumonia Emergency Response Epidemiology Team, 2020) and patients with severe COVID-19 may also develop neurological complications called Neuro-COVID (Heming et al., 2021). RNA sequencing is a very recently developed NGS methodology for the whole transcriptome or single-cell transcriptomics approaches (Liu and Di, 2020) and is broadly used to explore biological, cellular, and molecular processes implicated in COVID-19 infection (Liu T. et al., 2020; Liao et al., 2020; Zhou et al., 2020). Hence, either developing novel drug candidates or identifying suitable existing therapeutics for drug repurposing for COVID-19 and Neuro-COVID is essential to decrease the infection rate and control the COVID-19 pandemic and reduce the enormous economic burden on healthcare systems. Because the gene expression profiles of COVID-19 infection models can be used to decipher potential therapeutic targets that could be targeted by known drugs (Daamen et al., 2021), we used RNA-seq datasets from the COVID-19 infection models of NHBE cells, and the scRNA-seq datasets of immune cells isolated from the CSF of Neuro-COVID patients and analyzed using NGKD platforms to understand the disease-specific gene signatures and pathways and further uncover small molecules from both synthetic and natural sources that potentially reverse these diseases.

Here, we found that COVID-19 infection of NHBE cells activated upstream genes such as IL-17, TNF-alpha, STAT2, IRF9, and TLR-4. Biological processes such as humoral immune response, acute-phase response, and molecular functions such as cytokine activity, receptor regulator activity, signaling receptor activity, receptor-ligand activity, and chemokine activity were enriched in the COVID-infected cells. Importantly, the cytokine and cytokine receptor interaction and viral protein interaction with the cytokine and cytokine receptors were activated in COVID-infected NHBE cells. Cytokines are important for both innate and adaptive inflammatory host responses, cell differentiation, cell death, growth, repair and development, and cellular homeostasis (Pushparaj, 2019; Bahlas et al., 2020; Harakeh et al., 2020; Jafri et al., 2020; Pushparaj, 2020). Studies have shown that several circulating cytokines and chemokines such as TNFα, CXCL-10, IL-6, and IL-8 are differentially expressed during SARS-CoV-2 infection, and this cytokine/chemokine storm likely contributes to the poor prognosis of COVID-19 (Liu J. et al., 2020; Vaninov, 2020)**.** RNA sequencing analysis of cell and animal models of SARS-CoV-2 infection, blood, lung, and airway biopsies from COVID-19 patients showed inflammatory responses characterized by low levels of type I and III IFNs, increased interleukin-6 (IL-6), and a variety of chemokines (Blanco-Melo et al., 2020; Daamen et al., 2021). The spike protein (S protein) of SARS-CoV-2 is essential for the attachment between the coronavirus and hACE2 surface receptor through its receptor-binding domain (RBD) (Lan et al., 2020) and is proteolytically activated by human proteases, thus helping the coronavirus to enter the host cells (Shang et al., 2020). A recent study showed that hACE2 was stimulated by IFN in human airway epithelial cells (Ziegler et al., 2020) and thus helps in the entry of SARS-CoV-2 into host cells.

SARS-CoV-2 and other coronaviruses have developed different mechanisms to avoid detection and subsequent destruction by copying and repurposing cytokine and cytokine receptor genes in the host (Heimfarth et al., 2020; Choudhary et al., 2021). COVID-19 induced cytokines and cytokine receptors, chemokines, and other specific cytokine receptors and binding proteins may subvert and alter the host cytokine networks (Choudhary et al., 2021). Here, the COVID-19-induced cytokines, cytokine receptors, receptor-binding proteins, and chemokines may stimulate or prevent cytokine signaling and may significantly alter various facets of host immunity. In addition, Daamen et al. (2021) found that COVID-19 pathogenesis was driven by highly inflammatory myeloid-lineage cells with distinct transcriptional signatures and the absence of cytotoxic cells in the lungs, leading to reduced viral clearance.

Heming et al. (2021) stated that lumbar puncture to obtain immune cells from COVID-19 patients without neurological manifestations as controls was not ethically permitted for scientific purposes. Since IIH is a benign disorder associated with high pressure in the brain, the immune cells derived from the CSF of patients with IIH were used as controls to compare Neuro-COVID. The cluster of differentiation molecule 81 (CD81) is one of the commonly regulated genes in the meta clusters of immune cells from the CSF of patients with Neuro-COVID and belongs to the tetraspanin superfamily, which has been shown to regulate viral entry, viral replication, infectivity, and virion exit of different types of viruses (Benayas et al., 2020). Therefore, it is essential to investigate the importance of CD81 in patients with COVID-19 and Neuro-COVID. One of the upstream genes activated in TcMeta cluster, Cell cycle division 37 (CDC37), a heat shock protein 90 (HSP90) cochaperone that could play an important role in the pathogenesis of Neuro-COVID. COVID-19 progression to a systemic disease could be associated with HSP-related molecular mimicry autoimmune phenomena (Cappello et al. 2020; Kasperkiewicz, 2021). It was postulated that Hsp90 inhibition could also be a potential treatment option for cytokine storm-mediated acute respiratory distress syndrome in COVID-19 patients (Kasperkiewicz, 2021). Recently, Wyler et al. (2021) identified HSP90 as a target for COVID therapy based on transcriptomic profiling of SARS-CoV-2 infected human cell lines.

Interestingly, the top five differentially expressed pathways identified based on iPathwayGuide analysis of immune cell meta clusters from Neuro-COVID patients were malaria, African trypanosomiasis, cocaine addiction, Parkinson’s disease, and leukocyte transendothelial migration. Studies have shown a potential link between the presentation of malaria and COVID-19. The opposite relationship between COVID 19 and malaria has been suggested to be linked with the wide use of antimalarial drugs, including hydroxychloroquine (HCQ) and chloroquine (CQ), in countries that are endemic to malaria (Hussein et al., 2020).

There are many types of COVID-19 vaccines currently available for prophylaxis, and many are under development (Mandolesi et al., 2021). Several therapeutics are available based on WHO guidelines to treat the complications of COVID-19 and related complications (Lamontagne et al., 2021); however, these therapeutics are not specifically designed for the treatment of COVID-19 and its related complications such as Neuro-COVID, and their efficacies substantially differ across the globe and are not very effective in ameliorating disease severity (Surnar et al., 2020). In this study, we utilized NGKD platforms such as iPathwayGuide, L1000FWD, and L1000CDS2 tools to identify promising druggable molecules based on their *in silico* potential to reverse gene signatures induced by COVID-19 and Neuro-COVID. We found that camptothecin, importazole, and withaferin A had insufficient signaling or gene signatures (or absent) in COVID-19 infected NHBE cells. Based on L1000CDS2 analysis, trichostatin A, a histone deacetylase inhibitor, mildly inhibited the ACE receptors (Takahashi et al., 2021), and narciclasine and camptothecin are some of the top small molecules that reverse the gene signatures in Neuro-COVID vs. IIH immune datasets. In addition, a comparative analysis of the Neuro-COVID vs. IIH immune cell meta cluster datasets showed that JQ1 had insufficient signaling (or absence).

The GSEA preranked analysis calculates if *a priori* defined sets of genes display statistically significant enrichment at either end of the ranking (Subramanian et al., 2005). The gene signature potentially reversed by withaferin A in SARS-CoV-2 NHBE vs. Mock-NHBE based on preranked GSEA involved in various biological, molecular, and cellular processes, including viral genome replication (GO:0019079), modulation of the process of other organisms involved in symbiotic interactions (GO:0051817), and positive regulation of translational initiation (GO:0045948). The gene signature potentially reversed by importazole in SARS-CoV-2 NHBE vs. Mock-NHBE based on preranked GSEA involved in various biological, molecular, and cellular processes, including regulation of single-stranded viral RNA replication via a double-stranded DNA intermediate (GO:0045091). The gene signature potentially reversed by withaferin A in Neuro-COVID vs. IIH-TcMeta based on preranked GSEA involved in various biological, molecular, and cellular processes, including regulation of type 1 interferon production (GO:0032479) and interferon signaling (R-HSA-913531). The gene signature potentially reversed by withaferin A in Neuro-COVID vs. IIH-TcMeta based on preranked GSEA involved in various biological, molecular, and cellular processes, including regulation of type 1 interferon production (GO:0032479) and interferon signaling (R-HSA-913531). The gene signature potentially reversed by narciclasine in Neuro-COVID vs. IIH-TcMeta based on preranked GSEA involved in negative regulation of viral entry into host cells (GO: 0046597), PDGFR beta signaling pathway (PID-M186), EGF/EGFR signaling pathway (WP437), VEGFA/VEGFR2 signaling pathway (WP3888), and positive regulation of cell migration (GO:0030335). The gene signatures upregulated by withaferin A (GNLY CST7 PPIB TSPO BCL2 S100A10 GSTP1), (S100A10, TSPO, PPIB, HLA-DQB1, BCL2, GSTP1, EDF1, and FLOT1), and S100A9, S100A8, TSPO, and HOMER3) were positively enriched in Neuro-COVID. Granulysin (GNLY) is a member of the saposin-like protein (SAPLIP) family, is located in the cytotoxic granules of T-cells and NK cells, is released on antigen stimuli, and has antimicrobial activity. The S100 genes include 13 members and have antibacterial and antifungal properties (Crinier et al., 2018).

Our L1000FWD analyses showed that withaferin A was the top natural product that reverses the signature of Neuro-COVID in all the meta clusters of immune cells from the CSF of Neuro-COVID patients. Withaferin A is a component of Withania somnifera (*ashwagandha or Indian ginseng*) (Srivastava et al., 2020). W. somnifera has been used in traditional medicine as an antioxidant, antianxiety, anti-inflammatory, antibacterial, aphrodisiac, and herbal tonic for general health (Sood et al., 2018). The active ingredients include withanolides, saponins, alkaloids, and steroidal lactones. *In vitro* studies have shown that ashwagandha has neuroprotective, cardioprotective, immunomodulating, and anticancer properties (Singh et al., 2021).

Adjunctive treatment with ashwagandha improved symptoms and stress in patients with schizophrenia, offering beneficial effects on cognitive function in patients with bipolar disorder and improves balance in patients with progressive degenerative cerebral ataxias (Sood et al., 2018; Singh et al., 2021). It was recently shown that withanolides present in ashwagandha possess anti-COVID-19 properties**,** and these compounds exhibit good absorption and transport kinetics with no related mutagenic or adverse effects (Srivastava et al., 2020). Withaferin A was predicted to bind and stably interact with the binding site of TMPRSS2, similar to its known inhibitor, camostat mesylate (Kumar et al., 2020). Camostat was found to reduce SARS-CoV-2 infection in TMPRSS2 expressing Vero cells (Hoffmann et al., 2020). David et al., 2021 in their MedRixv preprint showed that a common variant of TMPRSS2 protects against COVID-19. *In silico* screening of several phytochemicals identified that Withanone, one of the constituents of ashwagandha, showed a potential inhibition of ACE2 (Balkrishna et al., 2021). Additionally, Ghosh et al. (2021) used molecular dynamic simulations and pharmacophore modeling approaches to predict the highly potent small-molecule derivative of withaferin A that potentially inhibits SARS-CoV-2 protease (Mpro), a favorable future therapeutic against COVID-19.

Recent studies have demonstrated the antiviral properties of narciclasine, an alkaloid found in various Amaryllidaceae species, and camptothecin, a topoisomerase inhibitor first isolated from the stem of *Camptotheca acuminata* (used in Chinese traditional medicine) against SARS-CoV-2 (Huang C.-T. et al., 2020; Mamkulathil Devasia et al., 2021). However, importazole, an inhibitor of importin-β transport receptors, and other small molecules identified to reverse COVID-19-induced gene signatures need to be further explored because developing effective therapeutics is essential to control the COVID-19 pandemic (Surnar et al., 2020).

In conclusion, the present study unravels a rapid approach to using high-throughput RNA sequencing technologies coupled with NGKD platforms to decipher specific drugs and small molecules derived either synthetically or from natural sources for the amelioration of COVID-19 related disease pathologies such as Neuro-COVID. Further studies are warranted to validate the small molecules identified in our study using *in vitro* and *in vivo* model systems of COVID-19 and Neuro-COVID to determine their mechanism(s) of action followed by suitable clinical trials to confirm the efficacy and safety for possible therapeutic intervention for COVID-19-related disease pathologies.

## Data Availability

Publicly available datasets were analyzed in this study. This data can be found here: Gene Expression Omnibus GSE147507 and GSE163005.

## Supplementary Material

Supplementary Table S1 The differentially regulated pathways in the meta clusters of immune cells from patients with Neuro-COVID are provided in Supplementary Table S1.

Supplementary Table S2 The upstream genes activated in TcMeta, DCMeta, and monoMeta clusters are listed in Supplementary Table S2.

Supplementary Datasheet S1 The genes enriched in GSEA preranked analysis of SARS CoV2-NHBE vs. Mock-NHBE against the gene signatures of camptothecin, importazole, and withaferin A are provided in Supplementary Datasheet S1.

Supplementary Datasheet S2 The genes enriched in GSEA preranked analysis of Neuro-COVID vs. IIH comparison against the gene signatures of withaferin A, camptothecin, trichostatin A, narciclasine, and JQ1 are provided in Supplementary Datasheet S2.

Supplementary Figure S1 **(A)** Hierarchically clustered interactive heatmaps were generated using the Clustergrammer web tool for visualizing and analyzing high-dimensional RNASeq data (NHBE-SARS CoV2 vs NHBE-Mock). **(B)** Principal Component Analysis (PCA) was applied to identify global patterns in high-dimensional RNASeq datasets **(C)** Volcano plot was generated using transformed gene fold changes using log2 and displayed on the x-axis **(D)** MA plot was based on average gene expression which was calculated using the mean of the normalized gene expression values and displayed on the x-axis.

Supplementary Figure S2 GSEA Preranked analysis was performed to decipher the potential gene signatures differentially regulated (Combined, Downregulated, and Upregulated) by camptothecin, importazole, and withaferin A using the RNK file generated from DEGs of SARS CoV-NHBE vs Mock-NHBE comparison. **(A)** The enrichment of gene signature (Signature_ID: CPC002_PC3_24H: BRD-A30437061:10.0) differentially regulated by camptothecin, **(B)** the enrichment of gene signature (Signature ID: CPC006_A375_24H: BRD-A02481876:60.0) differentially regulated by importazole and **(C)** the enrichment of gene signature (Signature ID: CPC014_VCAP_6H: BRD-A52193669:10.0) differentially regulated by withaferin A in SARS CoV2-NHBE cells.

Supplementary Figure S3 GSEA Preranked analysis was performed to decipher the potential gene signatures differentially regulated (Combined, Downregulated, and Upregulated) by withaferin A using the RNK file generated from DEGs of Neuro-COVID vs IIH (TcMeta, DCMeta, and monoMeta) comparisons. The enrichment of gene signature (Signature ID: CPC014_VCAP_6H: BRD-A52193669:10.0) differentially regulated by withaferin A in **(A)** Neuro-COVID vs IIH-TcMeta **(B)** Neuro-COVID vs IIH-DCMeta, and **(C)** Neuro-COVID vs IIH-monoMeta.

Supplementary Figure S4 GSEA Preranked analysis was performed to decipher the potential gene signatures differentially regulated (Combined, Downregulated, and Upregulated) by camptothecin using the RNK file generated from DEGs of Neuro-COVID vs IIH (TcMeta, DCMeta, and monoMeta) comparisons. The enrichment of gene signature (Signature ID: CPC002_PC3_24H: BRD-A30437061:10.0) differentially regulated by camptothecin in **(A)** Neuro-COVID vs IIH-TcMeta **(B)** Neuro-COVID vs IIH-DCMeta, and **(C)** Neuro-COVID vs IIH-monoMeta.

Supplementary Figure S5 GSEA Preranked analysis was performed to decipher the potential gene signatures differentially regulated (Combined, Downregulated, and Upregulated) by trichostatin A using the RNK file generated from DEGs of Neuro-COVID vs IIH (TcMeta, DCMeta, and monoMeta) comparisons. The enrichment of gene signature (Signature ID: CPC012_A375_6H: BRD-K68202742:10.0) differentially regulated by trichostatin A in **(A)** Neuro-COVID vs IIH-TcMeta **(B)** Neuro-COVID vs IIH-DCMeta, and **(C)** Neuro-COVID vs IIH-monoMeta.

Supplementary Figure S6 GSEA Preranked analysis was performed to decipher the potential gene signatures differentially regulated (Combined, Downregulated, and Upregulated) by narciclasine using the RNK file generated from DEGs of Neuro-COVID vs IIH (TcMeta, DCMeta, and monoMeta) comparisons. The enrichment of gene signature (Signature ID: CPC006_HA1E_24H: BRD-K06792661:10.0) differentially regulated by narciclasine in **(A)** Neuro-COVID vs IIH-TcMeta **(B)** Neuro-COVID vs IIH-DCMeta, and **(C)** Neuro-COVID vs IIH-monoMeta.

Supplementary Figure S7 GSEA Preranked analysis was performed to decipher the potential gene signatures differentially regulated (Combined, Downregulated, and Upregulated) by JQ1 using the RNK file generated from DEGs of Neuro-COVID vs IIH (TcMeta, DCMeta, and monoMeta) comparisons. The enrichment of gene signature (Signature ID: LJP008_A549_24H: BRD-K54606188:10) differentially regulated by JQ1 in **(A)** Neuro-COVID vs IIH-TcMeta **(B)** Neuro-COVID vs IIH-DCMeta, and **(C)** Neuro-COVID vs IIH-monoMeta.
