# Supplementary figures and images for: Identification of Novel Gene Signatures using Next-Generation Sequencing Data from COVID-19 Infection Models: Focus on Neuro-COVID and Potential Therapeutics
Source: Front Pharmacol. 2021 Aug 31;12:688227. doi: 10.3389/fphar.2021.688227 (PMC8438179; doi:10.3389/fphar.2021.688227)

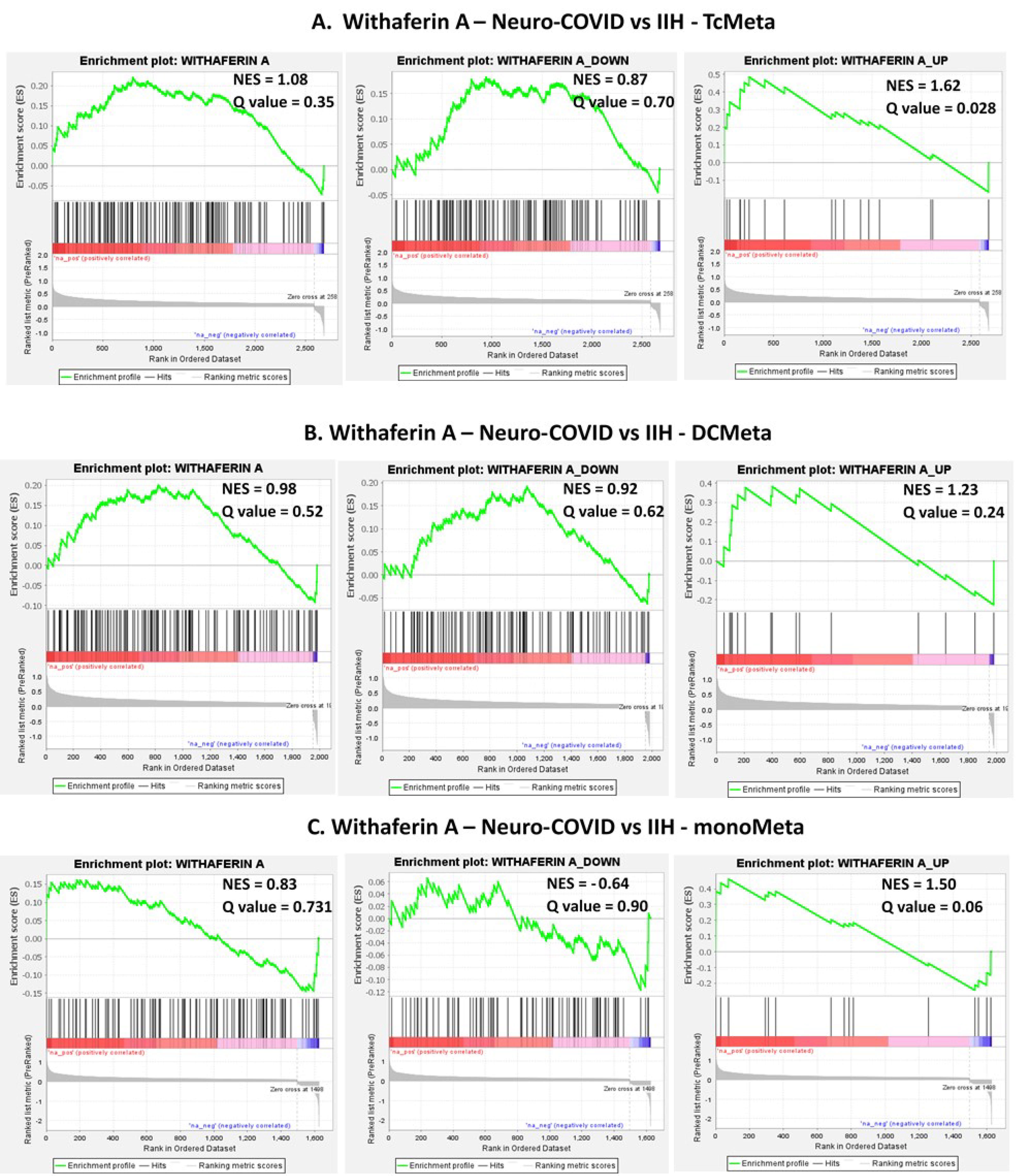

Supplement: Supplementary file 2 [file Image3.JPEG]

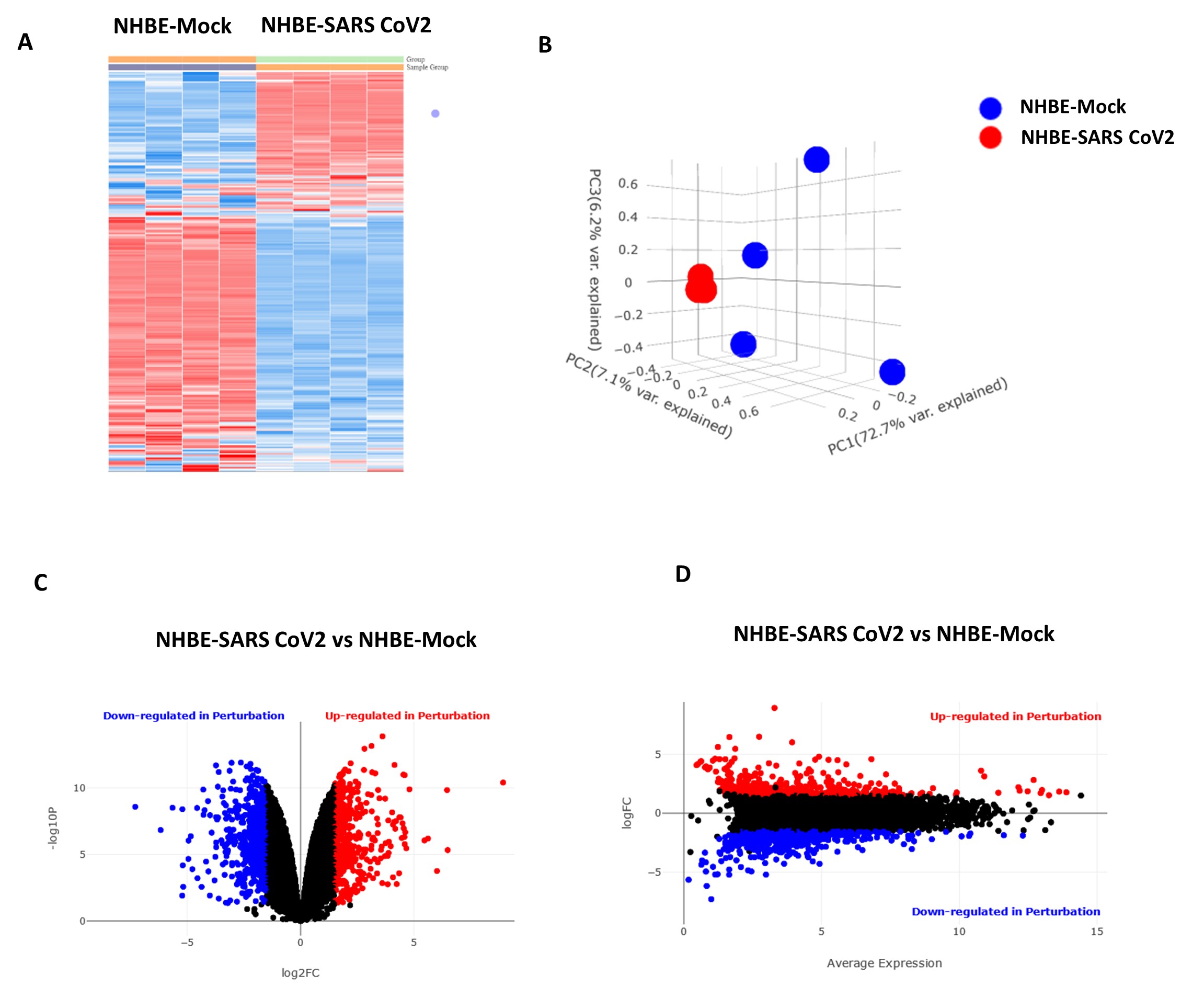

Supplement: Supplementary file 3 [file Image1.JPEG]

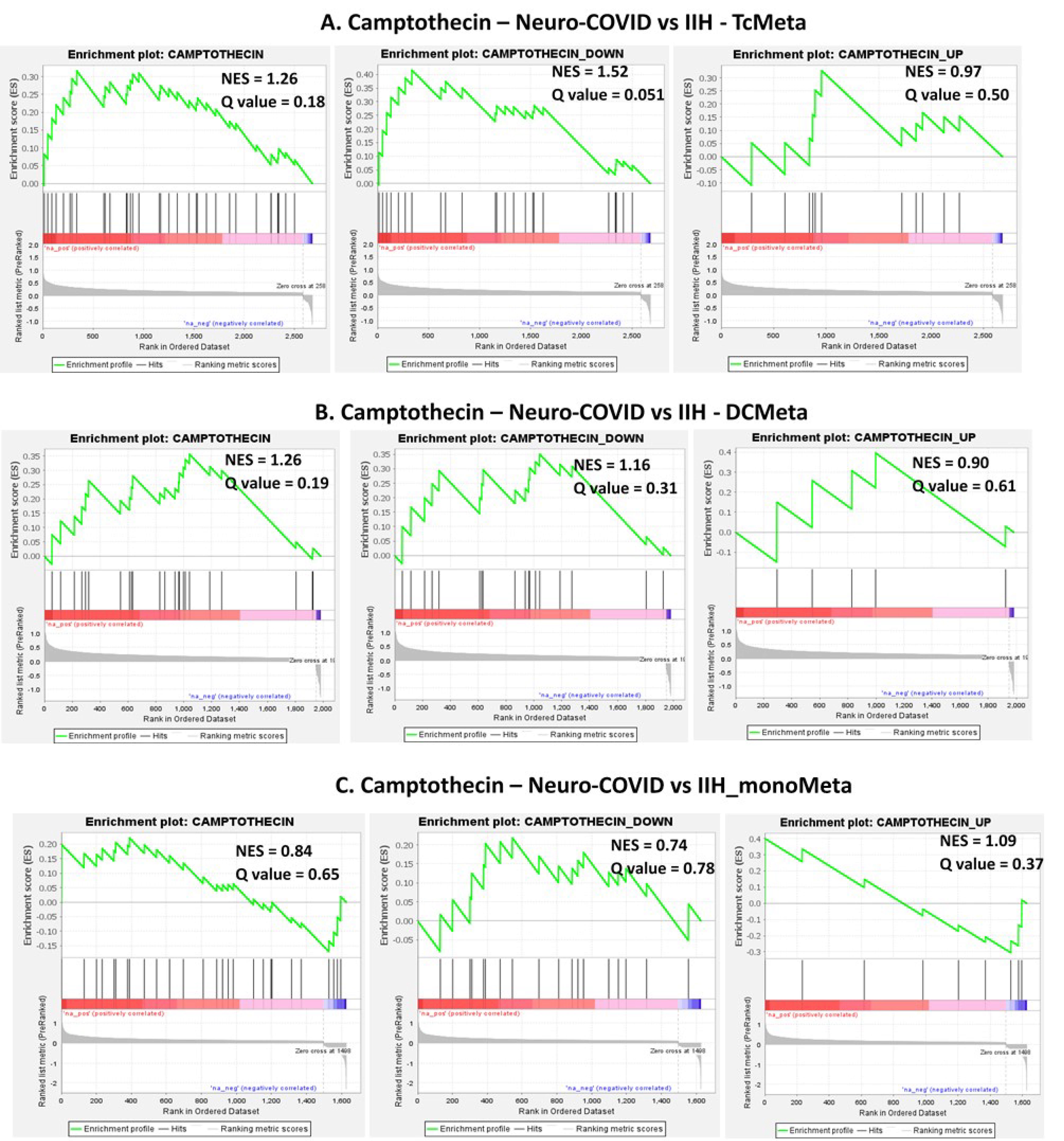

Supplement: Supplementary file 4 [file Image4.JPEG]

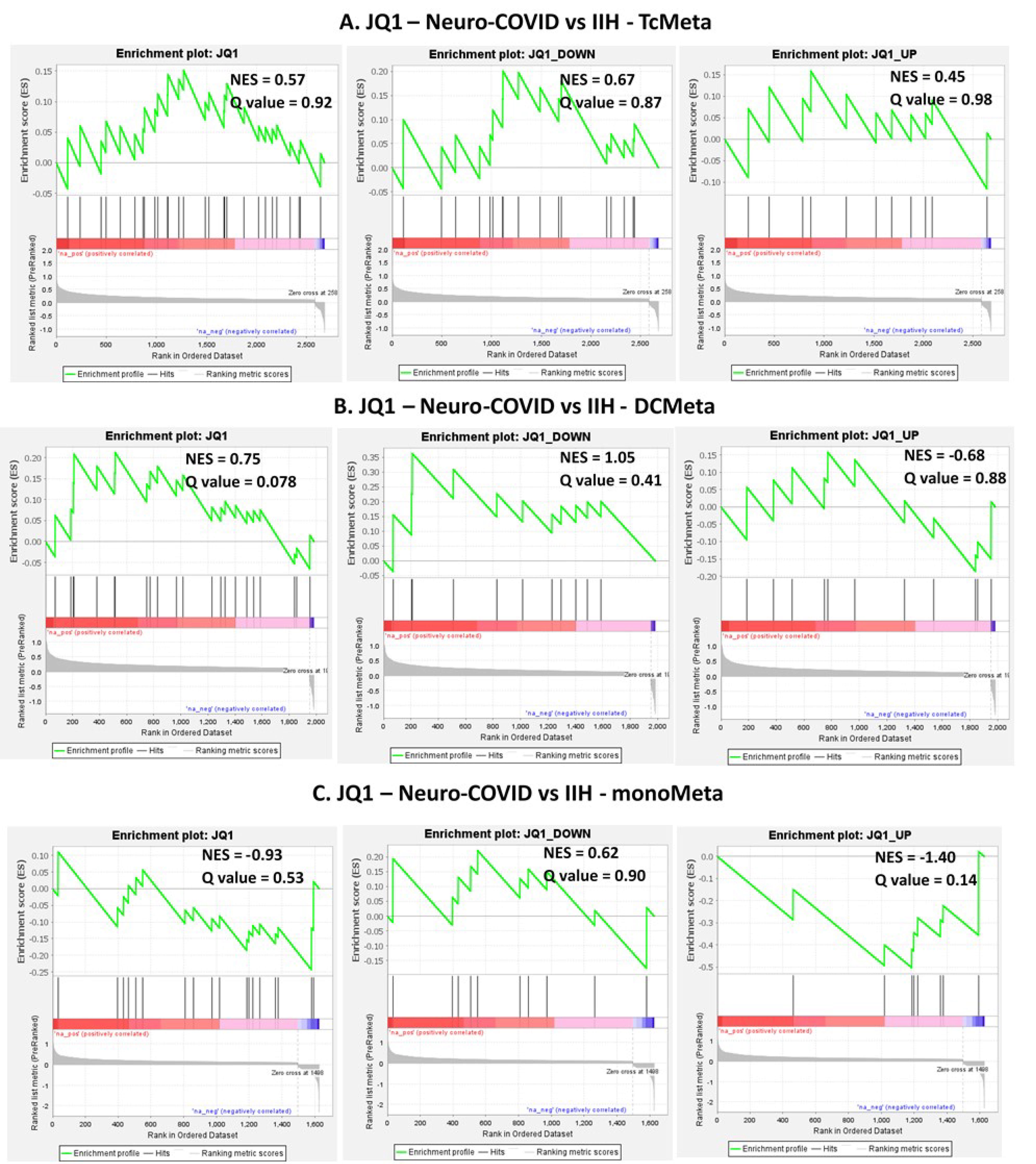

Supplement: Supplementary file 5 [file Image7.JPEG]

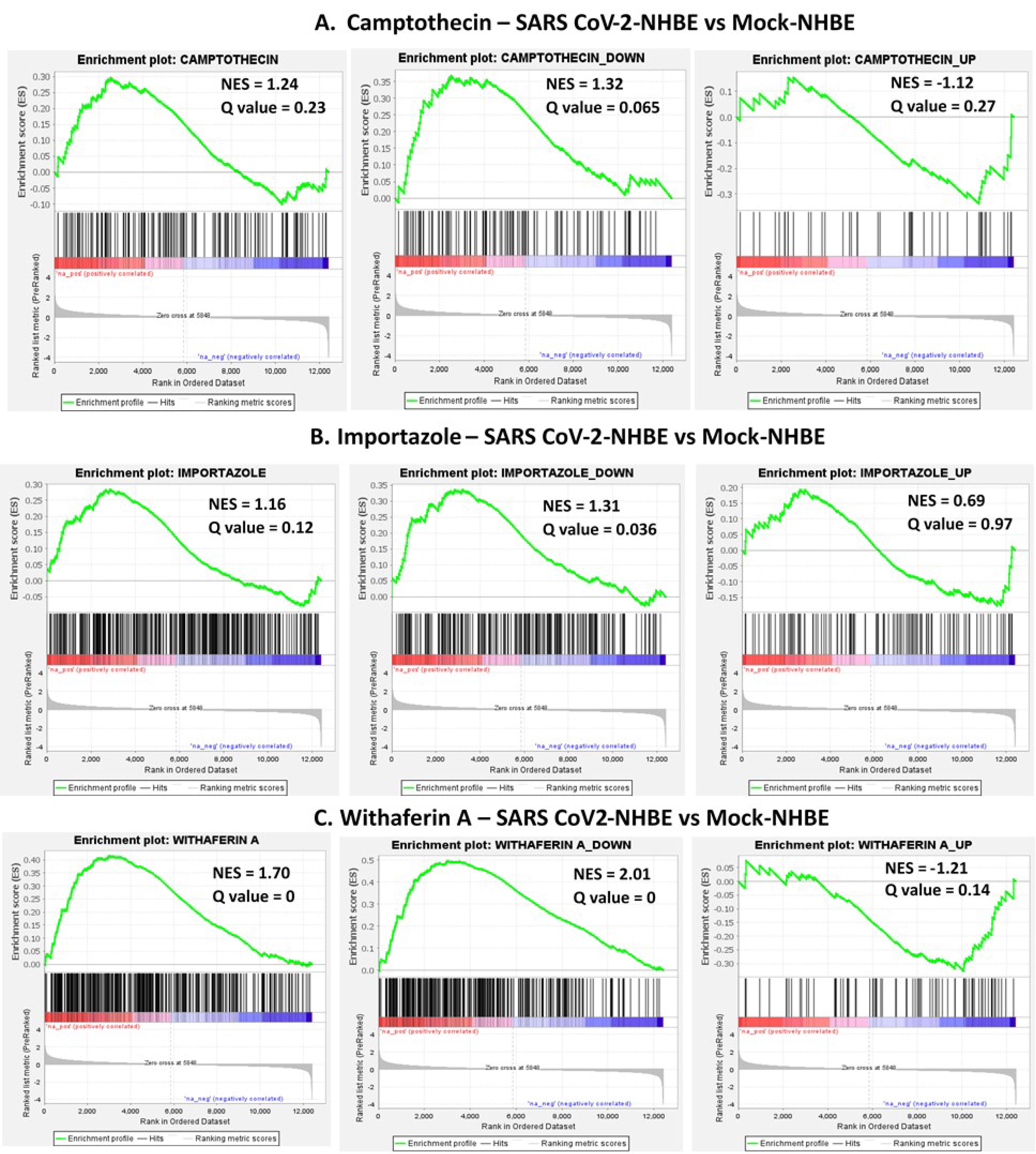

Supplement: Supplementary file 6 [file Image2.JPEG]

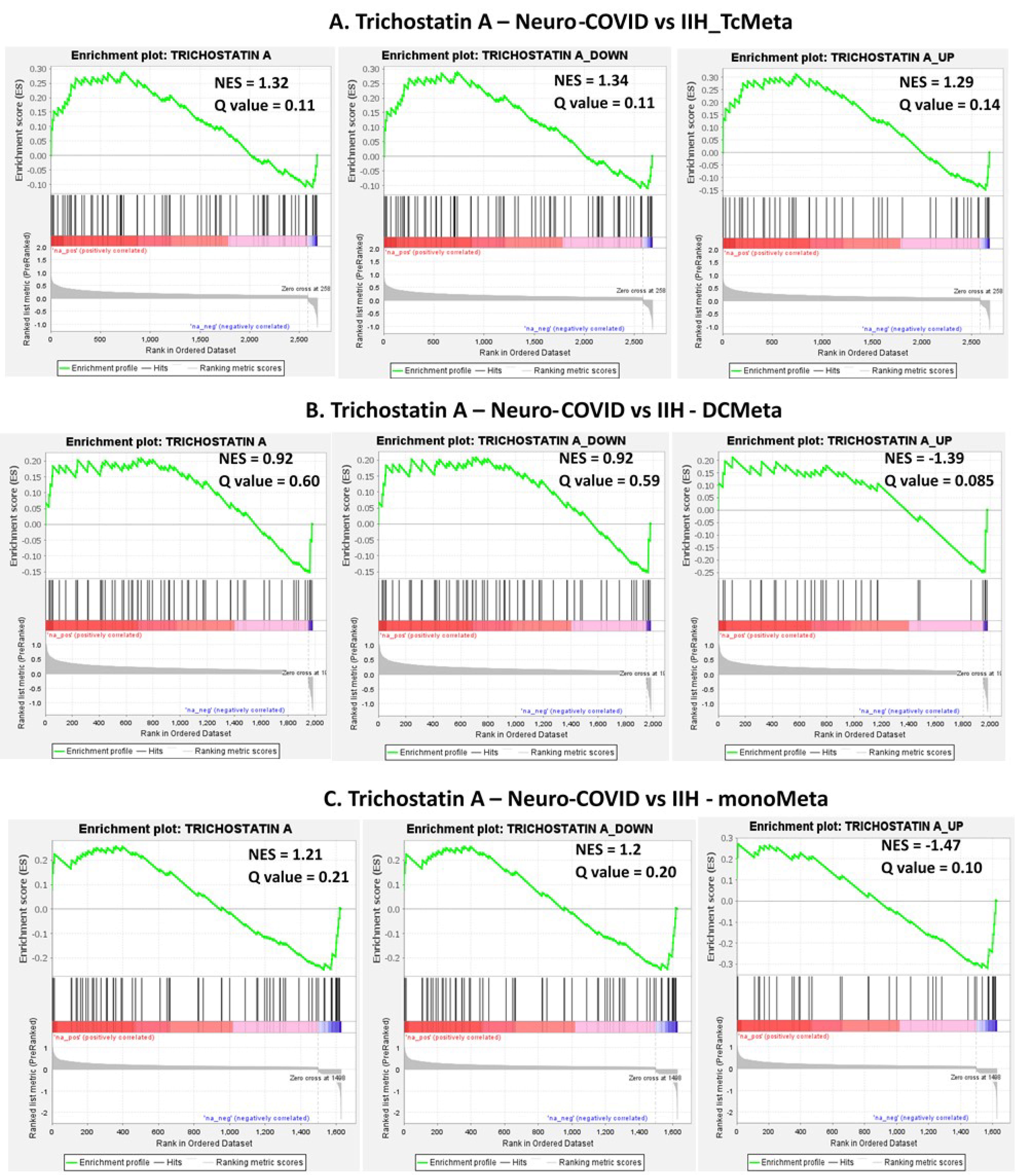

Supplement: Supplementary file 7 [file Image5.JPEG]

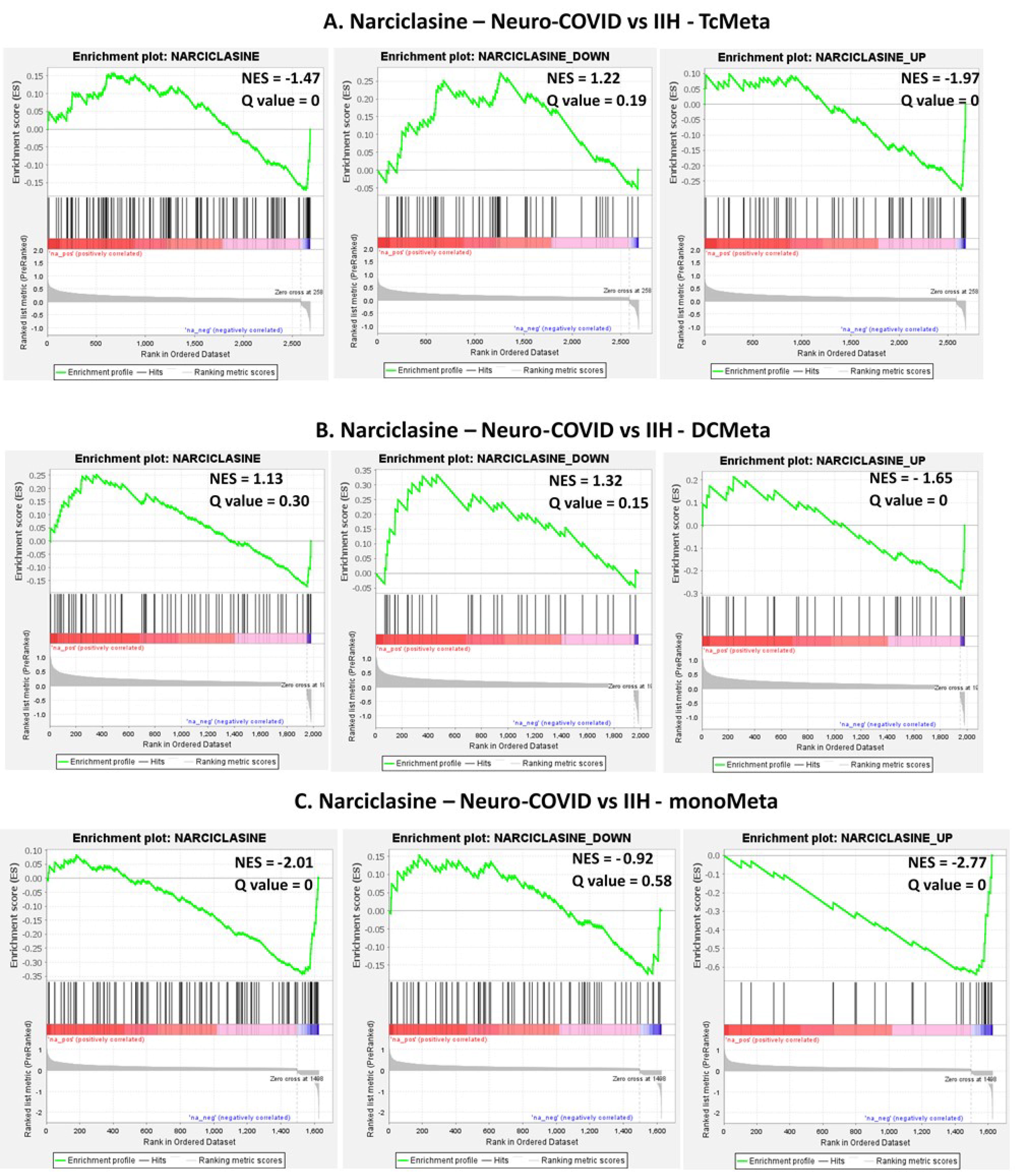

Supplement: Supplementary file 9 [file Image6.JPEG]
